# The Regulatory Mendelian Mutation score for GRCh38

**DOI:** 10.1093/gigascience/giad024

**Published:** 2023-04-21

**Authors:** Max Schubach, Lusiné Nazaretyan, Martin Kircher

**Affiliations:** Exploratory Diagnostic Sciences, Berlin Institute of Health at Charité–Universitätsmedizin Berlin, 10117 Berlin, Germany; Exploratory Diagnostic Sciences, Berlin Institute of Health at Charité–Universitätsmedizin Berlin, 10117 Berlin, Germany; Exploratory Diagnostic Sciences, Berlin Institute of Health at Charité–Universitätsmedizin Berlin, 10117 Berlin, Germany; Institute of Human Genetics, University Medical Center Schleswig-Holstein, University of Lübeck, 23562 Lübeck, Germany

**Keywords:** variant prediction, machine learning, web service, Mendelian disease, noncoding score, rare variant analysis, imbalanced data

## Abstract

**Background:**

Genome sequencing efforts for individuals with rare Mendelian disease have increased the research focus on the noncoding genome and the clinical need for methods that prioritize potentially disease causal noncoding variants. Some tools for assessment of variant pathogenicity as well as annotations are not available for the current human genome build (GRCh38), for which the adoption in databases, software, and pipelines was slow.

**Results:**

Here, we present an updated version of the Regulatory Mendelian Mutation (ReMM) score, retrained on features and variants derived from the GRCh38 genome build. Like its GRCh37 version, it achieves good performance on its highly imbalanced data. To improve accessibility and provide users with a toolbox to score their variant files and look up scores in the genome, we developed a website and API for easy score lookup.

**Conclusions:**

Scores of the GRCh38 genome build are highly correlated to the prior release with a performance increase due to the better coverage of features. For prioritization of noncoding mutations in imbalanced datasets, the ReMM score performed much better than other variation scores. Prescored whole-genome files of GRCh37 and GRCh38 genome builds are cited in the article and the website; UCSC genome browser tracks, and an API are available at https://remm.bihealth.org.

## Findings

### Introduction

The Regulatory Mendelian Mutation (ReMM) score predicts the potential pathogenicity of noncoding variants [1]. It is specifically designed for highly imbalanced datasets with an excess of neutral variants, which naturally occurs in whole-genome sequencing of probands with Mendelian disorders because only a small number of variants are expected to be causal among thousands of observed variants. The original score was constructed on the human reference genome build GRCh37/hg19. Nowadays, the standard for sequencing projects in clinic and research is the updated reference genome GRCh38/hg38. It contains new sequences at nearly 100 assembly gaps and reduces unresolved bases at about 3% of the genome [2]. Often, coordinate liftovers are performed between builds, but they are limited to well-characterized regions in both genome builds and may be insensitive to changes in the exact sequence. In addition to the advantages of an updated reference genome [3, 4], new annotations may primarily support GRCh38. This establishes a need for an update of the ReMM score, and we present a version developed particularly for GRCh38. Further, we update the ReMM score for GRCh37 by including feature updates and improving its handling of missing values. We show that the score has superior performance on imbalanced datasets compared to competing approaches and the most frequently used scores in the field. Finally, we provide a webserver and API for scoring VCF files, single-variant lookups, or range lookups.

### Methods

#### Training set labels and hyperparameters

The ReMM score is based on an imbalance-aware machine learning algorithm, hyperSMURF [5], trained from known pathogenic noncoding variants of Mendelian disorders and a set of putatively benign variants. As a pathogenic set, we use 406 hand-curated variants already used in the prior ReMM version [1], reciprocally lifted to GRCh38 using UCSC liftOver (RRID:SCR_018160) v377 [6] and validated for identical allelic sequences. The proxy-benign set includes around 14 million of human lineage–derived sequence alterations [7], which we filtered to noncoding sequences using Jannovar v0.36 [8] and RefSeq (RRID:SCR_003496) [9]. Restricting variants to noncoding only removes a small proportion of variants (0.7% and 1% for GRCh37), and the high imbalance with the pathogenic variant set is similar on both genome builds (14.8 million and 13.9 million proxy-benign variants for GRCh37 and GRCh38, respectively). Therefore, we kept parameters for hyperSMURF model training as determined in [1] (Supplementary Table S1).

#### Imbalance-aware model training

The hyperSMURF algorithm applies a special sampling technique essential for the highly imbalanced data of human pathogenic variants [1, 5]. The minority class (for ReMM, the pathogenic variants) is oversampled based on the Synthetic Minority Over-sampling Technique (SMOTE) that creates synthetic examples using k-nearest neighbors rather than oversampling the data with replacement [10]. The majority class (proxy-benign set) is divided into *n* nonoverlapping partitions, which then are subsampled according to a ratio parameter. The minority class is oversampled by factor 2, and the majority class is undersampled by factor 3, which leads to the ration of pathogenic versus benign variants of 2–3 in a more balanced dataset with around 2,000 data points. However, each balanced dataset alone provides insufficient coverage of the large data space of the majority class. That is why hyperSMURF applies an ensemble method: it divides the dataset into 100 partitions, each containing all oversampled pathogenic and 1 partition of downsampled proxy-benign variants. On each partition, a random forest [11] is trained and the final pathogenicity score is the average over the 100 predictions. It ranges from 0 (not pathogenic) to 1 (pathogenic) and gives the probability values of a variant to belong to the pathogenic training data. Thus, the higher the score, the more likely that a variant at that position is pathogenic. We used parSMURF as an implementation of hyperSMURF, a fast and highly scalable model training tool based on random forest algorithms [12].

#### Cytogenic band-aware cross-validation

Genomic data are confounded by local correlation of annotations (i.e., gnomically proximal variants tend to be more similar in their annotation results than random variants). Further, known pathogenic variants are not distributed evenly across the genome (e.g., due to selection bias, shared identification, available validation assays) but rather cluster around certain well-studied genes and share certain molecular functions or properties. When not accounted for, learners might infer superior hold-out performance because of genomic proximity of variants. To handle the local correlation structure in the genome, we apply 10-fold cytogenic band-aware cross-validation (CV) [1]. This is a stratified CV approach where each cytoband of the genome is associated to 1 out of 10 folds. Folds are assigned to have a similar number of pathogenic variants, and cytobands without pathogenic variants are randomly assigned to a fold. Proxy-benign variants are considered in the folds of their associated bands. Thereby, gnomically proximal (i.e., same cytoband) pathogenic and proxy-benign variants are considered together, making it more challenging for the learner to discriminate between the 2 groups. Ten separate models are trained on 9 folds and validated on the 10th fold. Unbiased predictions of variants contained in the training set can be performed with the model that only used the variants in the validation fold, while other variants are reported by a general model trained on all input data.

#### Model features and imputation

Twenty-six selected features (see Supplementary Table S2) capture functional constraint and different sequence functions (sequence composition, epigenetics, conservation, population variance, and regulatory regions) of the genetic variants. The feature set was kept close to the original feature set of ReMM, but some were not available from the original databases or were updated. Some features have a high proportion of missing values, and the initial version of ReMM imputed all of them with zero. In genomics, a missing value often indicates an experimental signal that is too low to be measured, in line with this imputation. We have now identified some features (e.g., GC content or conservation scores) where the genome-wide average of the annotation is more appropriate and impute them differently in this version (see Supplementary Table S2). For missing *P* values, we use the value 1.

#### Availability of prescored files and scoring workflow

Prescored, block-gzip compressed and indexed whole-genome files [13] were generated to allow a fast scoring of variants as well as an easy integration into other software. Every genomic position was scored with a general ReMM model trained on all data (v0.4.hg19 and v0.4.hg38, respectively). To guarantee unbiased score usage (e.g., for performance benchmarks with other tools), we replaced the score of variants in the training set with cross-validated scores (see above). The training and scoring pipeline is implemented in snakemake, a workflow management system for reproducible and scalable analysis [14, 15].

#### ClinVar dataset

Version 2022–12-03 of NCBI ClinVar (RRID:SCR_006169) was downloaded on 19 December 2022. Variants were filtered for single-nucleotide changes with unambiguous clinical assertions of “pathogenic,” “likely pathogenic,” “likely benign,” and “benign.” The set was annotated using Jannovar as described above and filtered for noncoding effects. Variants overlapping the training dataset as well as mitochondrial single-nucleotide variants (SNVs) were excluded (remaining *n* = 946 likely pathogenic/pathogenic and *n* = 192,057 likely benign/benign).

#### Comparison with other scores

For performance comparison on the GRCh38 training set (CV results as described above) of ReMM v0.4.hg38 with other scores, prescored GRCh38 whole-genome files of CADD [7] version 1.6 were used to retrieve raw scores. ExPecto [16] and Sei [17] scores were computed using VCF files as described on their source code repositories [18, 19]. For ExPecto, the UCSC hg19 fasta reference file was replaced with hg38 to retrieve scores on the new genome build. Sei was run with the –hg38 option, respectively. The absolute mean and absolute maximum over all 218 outputs were used as final scores of Expecto. On the NCBI ClinVar set, we used GRCh37 whole-genome files of CADD v1.3, CADD v1.6. [7], ExPecto [16], and Sei [17] as described above but with the UCSC hg19 reference genome. LINSIGHT scores [20] were downloaded from its source code repository [21] in bigWig format and extracted using the pyBigWig package [22]. fathmm-MKL [23] and RegBase [24] were downloaded in VCF format from the respective source code repositories [25], and the scores were extracted using bcftools intersect [26]. The ncER v2 [27] BED file was downloaded from its dataset repository [28] using bedtools intersect to retrieve scores. ReMM scores for hg38 were included in the comparison by lifting the variant positions from the ClinVar set to the hg38 reference genome using UCSC liftOver (v377) [6] and extracting the corresponding ReMM v0.4.hg38 scores from the whole-genome file.

### Results

#### Performance of ReMM on GRCh38

**Figure 1:**
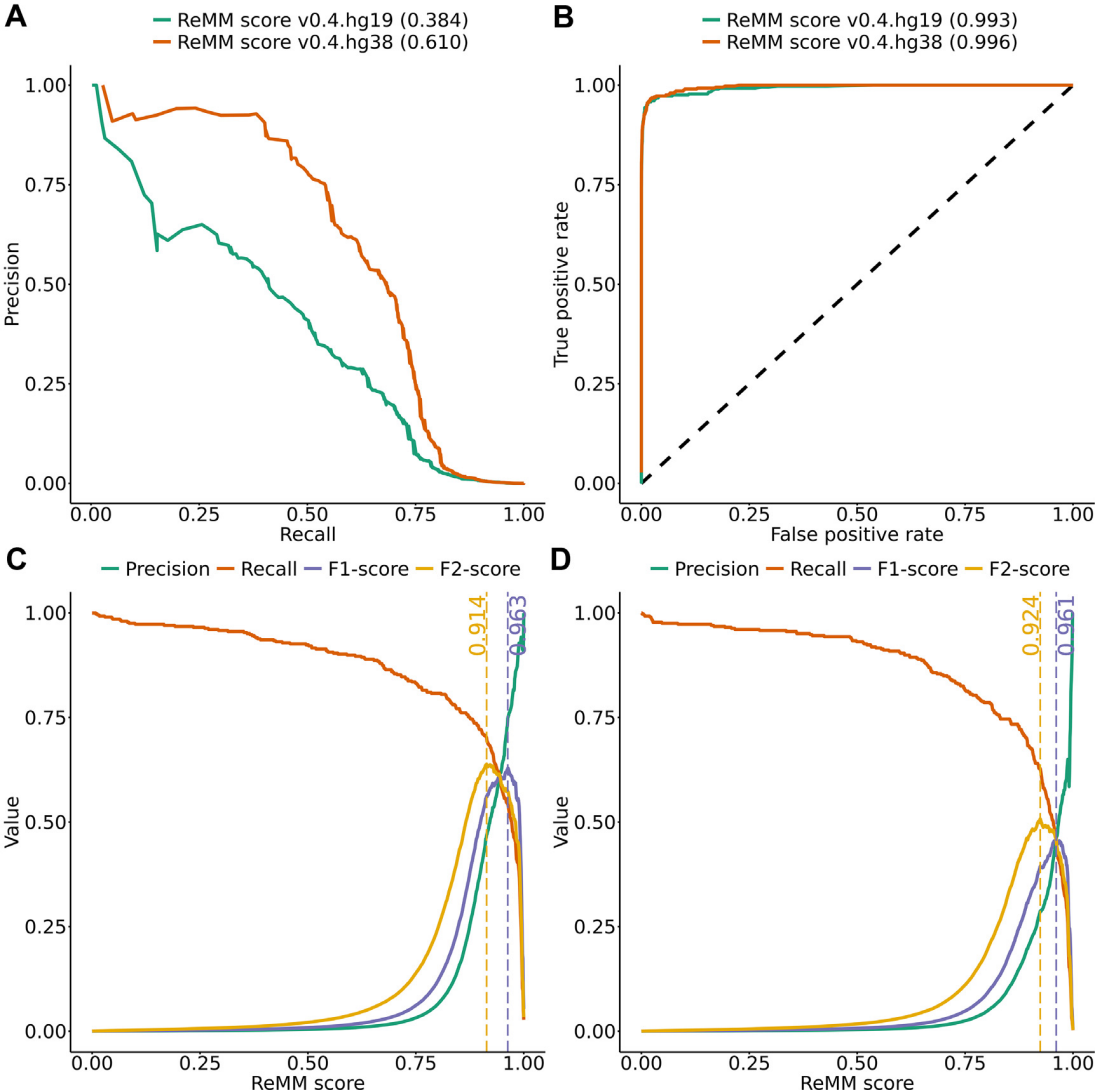
Precision-recall, ROC, and F1- and F2-score curves—performance metrics of ReMM v0.4.hg19 and v0.4.hg38 generated via 10-fold cytoband cross-validation. Precision-recall curves (A), ROC curves (B), and precision-recall, F1-score, and F2-score (y-axis) over different ReMM score thresholds (x-axis) for v0.4.hg38 (C) and v0.4.hg19 (D). Vertical lines denote the ReMM score with the maximum F1-score (yellow) and the maximum F2-score (purple). Area under the curve is shown in parentheses.

After 100 training cycles using different random seeds and 10-fold cytoband cross-validation, we achieved a performance with an average area under the precision recall curve (AUPRC) of 0.613 ± 0.005 (Supplementary Table S3). We randomly picked 1 model for the final scoring with an AUPRC of 0.610 (Fig. 1A, receiver operating characteristic [ROC] performance available in Fig. 1B).

Rather than using ReMM scores for ranking, some users choose to specify score thresholds for classifying into pathogenic and benign variants. Using a cutoff of 0.5 yields a good result in terms of retrieving known pathogenic noncoding variants (i.e., recall or true-positive [TP] rate), but the number of benign variants might be extremely large. For ReMM v0.4.hg38, recall is 92% (375 of 406) at a cutoff of 0.5 (Fig. 1C), but precision is close to zero with a high false-positive (FP) rate (86,507 of 13,911,061; FP rate = 0.006). The F1-score (harmonic mean of recall and precision) is highest at 0.963, resulting in a TP rate of 0.554 and an FP rate of 5.3e-6. Using the F2-score, we can give more weight to recall. Here, the optimal cutoff is 0.914, resulting in a TP rate of 0.702 and an FP rate of 2.3e-5. Analogous to NCBI ClinVar [29] pathogenic and likely pathogenic categories, we suggest using a ReMM score above the F1 threshold as weak computational evidence for “pathogenic” and a score above the F2 threshold and below the F1 threshold for “likely pathogenic.” For ReMM v0.4.hg19, these thresholds are 0.961 and 0.924 (Fig. 1D), respectively.

#### Correlation of scores and features

**Figure 2:**
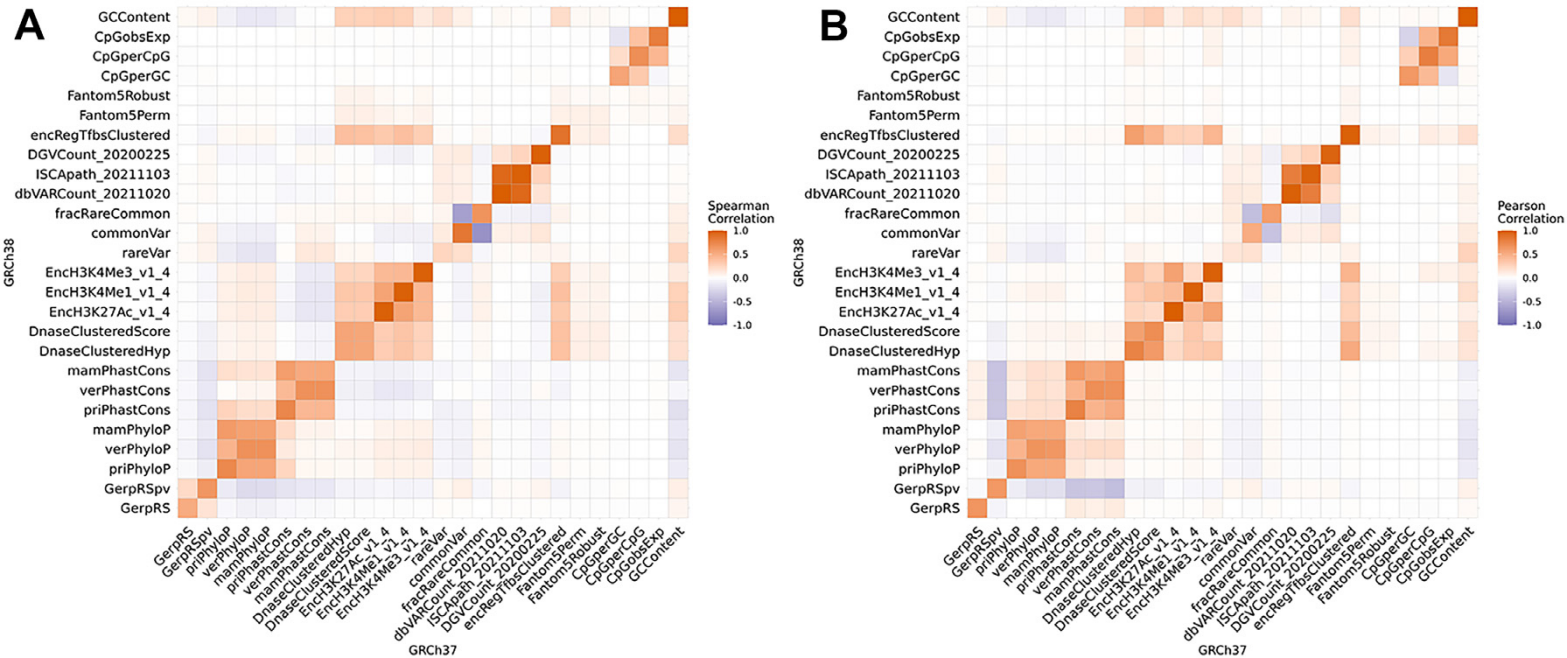
Correlation of feature values across genome builds—feature correlation between features of the GRCh37 (x-axis) and the GRCh38 (y-axis) genome builds. The left heatmap (A) shows Spearman correlation, and the right (B) shows Pearson correlation. Both plots show unexpectedly low correlations for some features along the diagonal. For example, the histone modification features (ENCODE) are lowly correlated as well as the enhancer features (FANTOM).

To compare both genome builds, we correlate ReMM scores from three genomic regions without assembly gap changes (DLK1, HBB, and PRDM9 loci) and >100,000 randomly sampled autosomal positions with successful reciprocal liftover (Supplementary Table S4). Here, ReMM scores are highly correlated between versions (Spearman and Pearson correlation between 0.7 and 0.8; Supplementary Table S4). We also used these regions and sites to explore the average feature correlation and find those to be similar, with the exception of 1 region (PRDM9), which is lower (Spearman correlation of 0.7 and Pearson correlation between 0.6 and 0.8; Supplementary Table S5). Further, we compare feature correlations between the genome builds directly on the training data (Fig.   2). As expected from the high sequence similarity between reference sequence versions, we see the highest correlation for sequence features, like GC content. Further, population variance features correlate well, with reduced correlation for the rare variant feature. This is likely due to spurious calls highly depending on the caller and the quality of the reference genome. We see the lowest correlation on the sparse Fantom5 regulatory element annotation data.

#### Imputing missing values

In previous ReMM versions, we used zero for missing values globally and trusted the nonlinearity of decision trees. Now, we use the average value of all defined positions for sequence and conservation features and one for *P* values (see Supplementary Table S2). With the new approach, we see that the average AUPRC increases slightly (0.005 for v0.4.hg19 and 0.009 for v0.4.hg38; Supplementary Table S6).

#### Feature importance

From the underlying Ranger random forest (RF) models [30], we retrieve feature importance using the Gini index. We averaged values over all 100 RFs in the model (Supplementary Table S7). In general, mean feature importance scores indicate contributions of all 26 features. No single feature stands out, and our broad feature categories are all represented with at least 1 highly ranked feature. We interpret this as evidence that features were carefully picked and biases avoided. Epigenetic features increased in importance for the GRCh38 model (average rank 16 vs. 19), which may be due to better mapping and processing of the underlying data. Fantom5 features are probably too sparse to receive high importance but might be relevant for some variants. Between genome builds, feature importance values are similar, and no significant changes are detected (*P* = 0.565, 2-sided rank-sum test). The replaced encRegTfbsClustered feature achieves a similar average Gini index (rank 6 on v0.4.hg19) as the previous numTFBSConserved feature (rank 4, data not shown).

#### Comparison to other scores

**Figure 3:**
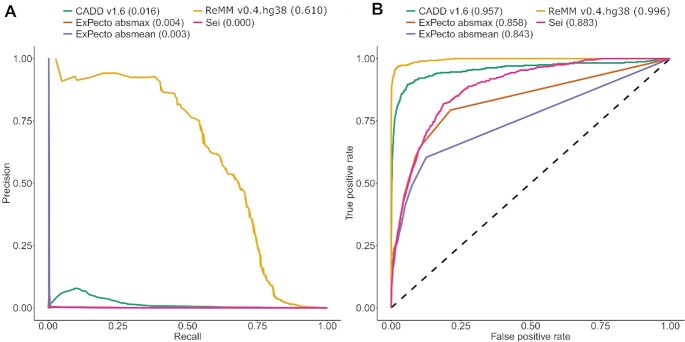
ROC and PR curves of ReMM, CADD, ExPecto, and Sei—PR curves (A) and ROC curves (B) of ReMM v0.4.hg38 (10-fold cytoband cross-validation scores) as well as CADD v1.6, ExPecto, and Sei on the GRCh38 training data. Area under the curve is shown in parentheses. ExPecto absmax is the maximum absolute value over all ExPecto outputs and ExPecto absmean the mean absolute value, respectively.

A number of different tools for scoring pathogenicity of noncoding variants exist [31]. However, most tools are still based on the GRCh37 genome build, which comes with the previously discussed drawbacks when scores are lifted to a new genome build [3, 4, 32]. To our knowledge, CADD, one of the most popular whole-genome scores, seems to be the only tool directly trained on training data and features derived from GRCh38. Nine years after the GRCh38 release, no other noncoding score is adapted to the new genome build. Also, more recent sequence-based tools, like ExPecto [16] or DeepSEA-Sei [17], are trained on the previous genome release. However, coordinate liftover can be avoided for those tools because predictions are solely based on sequence, and the sequence around variants from GRCh38 can be used directly. We compared performance of ReMM with CADD v1.6 GRCh38, Sei, and ExPecto on the GRCh38 imbalanced training data. The area under the precision-recall (PR) curve of ReMM substantially outperforms other methods (Fig. 3A), while the area under the ROC curve is above 0.8 for all tools (Fig. 3B). In the context of extremely unbalanced data, the area under the PR curve is more informative than the area under the ROC curve [33]. In these figures, the number of variants varies depending on how many were annotated with the respective tools. Specifically, ExPecto annotated only 7,299,993 out of the 14 million proxy-benign variants, probably due to missing transcripts close by. Therefore, its performance might be overestimated. In Supplementary Fig. S1, PR and ROC curves from the intersection of variants scored by all tools are shown (406 pathogenic and 7,299,993 proxy-benign variants), confirming that order and general result are stable despite the difference in the number of scored variants.

**Figure 4:**
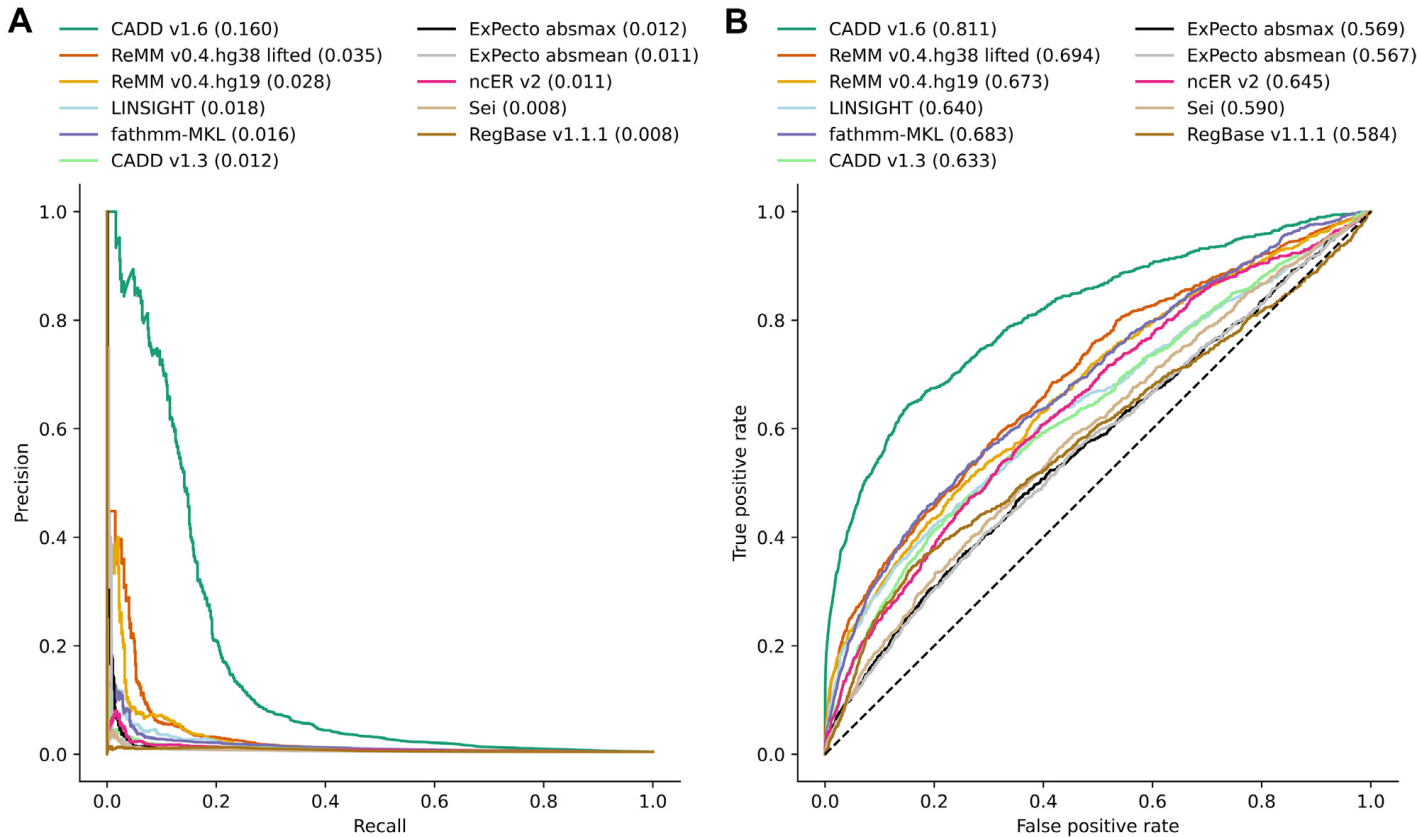
ROC and PR curve of noncoding NCBI ClinVar—PR curves (A) and ROC curves (B) of different pathogenicity scores on NCBI pathogenic/likely pathogenic and benign/likely benign variants in the noncoding genome (absent from the training set of ReMM). All scores are trained and available on GRCh37. For ReMM v0.4.hg38 lifted, we lifted the GRCh37 ClinVar positions using UCSC liftOver and looked up the corresponding ReMM v0.4.hg38 score. Area under the curve is shown in parentheses, and score names are sorted descending on the area under the PR curve. ExPecto absmax is the maximum absolute value over all ExPecto outputs and ExPecto absmean the mean absolute value, respectively.

Due to the very limited availability of noncoding scores on GRCh38, we compared ReMM on GRCh37 with multiple other scores and on a set of noncoding variants from NCBI ClinVar that do not overlap its training set. We only used variants where all scores were able to provide a prediction (869 pathogenic and 190,548 benign) and plotted PR and ROC curves (Fig. 4A,B). CADD v1.6 achieved the best performance in terms of AURPC (0.160) and area under the ROC curve (AUROC = 0.811), followed by the (liftover) GRCh38 version of ReMM (AUPRC = 0.035, AUROC = 0.694). Interestingly, CADD v1.3, a previous version that does not yet include features for intronic splice variants, has a much lower performance on the noncoding ClinVar dataset (AUPRC = 0.012, AUROC = 0.633). We therefore speculate that the performance boost in CADD v1.6 is due to the presence of many splice variants in the noncoding ClinVar dataset and the inclusion of specific splice scores, like SpliceAI [34] or MMSsplice [35], in recent CADD versions. All compared scores, excluding CADD v1.6, are not optimized for splicing effects. Further, ReMM's pathogenic training set does not contain splice variants, and we did not add specific splicing features with this update.

### Conclusion

The ReMM v0.4 score is a fully retrained noncoding score available for both the GRCh37 and GRCh38 genome builds. This fills the high need of supporting variant prioritization on the GRCh38 genome release, which is the *de facto* standard in research and routine diagnostics. Scores over the GRCh38 genome are highly correlated to the prior release with a performance increase for GRCh38 due to the better coverage of features. On imbalanced data (commonly observed in whole-genome sequencing of individuals affected with Mendelian disease), ReMM scores outperform other noncoding effect scores. However, our analysis of new noncoding ClinVar variants also highlights limitations when scores are applied to variants (here splice variants) missing from the training data or for which no specific model features were included. In summary, we established a reproducible and scalable framework for integration of new features or new training data for further development of ReMM. The prescored whole-genome files, UCSC genome browser annotation tracks [36], and a website provide fast access and easy usage of the ReMM score for researchers in all areas. With this release, tools like Genomiser [1] can now be run on the latest genome build, a highly demanded feature from the community.

## Availability of Supporting Source Code and Requirements

Project name: ReMM score

Project homepage: https://remm.bihealth.org

Operating system(s): Platform independent (website), Linux (workflow)

Programming language: Python, Java, C++, Bash

Other requirements: browser (website); conda, snakemake, parSMURF (workflow)

License: MIT License

RRID:SCR_023095

## Supplementary Material

giad024_GIGA-D-22-00232_Original_Submission

giad024_GIGA-D-22-00232_Revision_1

giad024_Response_to_Reviewer_Comments_Original_Submission

giad024_Reviewer_1_Report_Original_Submission Yan Guo -- 10/11/2022 Reviewed

giad024_Reviewer_1_Report_Revision_1 Yan Guo -- 2/27/2023 Reviewed

giad024_Reviewer_2_Report_Original_Submission Mulin Jun Li -- 11/18/2022 Reviewed

giad024_Reviewer_3_Report_Original_Submission Wyeth Wasserman -- 11/22/2022 Reviewed

giad024_Supplemental_File

## Data Availability

We precomputed ReMM scores for all sequence-resolved positions in the genome (GRCh37 and GRCh38 builds) and provide them on Zenodo [37] or on the ReMM website [38], where we enable fast and easy scoring of variants. Variants can be uploaded via a VCF file [39], or scores directly displayed with a single site or genomic range variant lookup. Usage of UCSC genome browser tracks [36] of ReMM scores is described on the same website. In addition, we provide a REST-API that allows tools and scripts to retrieve ReMM scores directly. Scoring on the website is available for both genome builds and all major ReMM versions. ReMM is registered at bio.tools (biotools:remm_score) and has a Research Resource Identification Initiative ID (RRID:SCR_023095). The snakemake workflow to generate features, train scores, and generate whole genome files is available on GitHub [40] or on WorkflowHub [14]. All supporting data are available in the *GigaScience* GigaDB database [41].

## Additional Files

**Supplementary Table S1**. Hyperparameters of ReMM training (used across all currently available versions).

**Supplementary Table S2**. Features used for training of the ReMM v0.4 score and their default for missing values. The description column contains the original source from where features were downloaded.

**Supplementary Table S3**. ReMM score v0.4 performance—area under the precision recall curve (AUPRC) and area under the receiver operating characteristic curve (AUROC) for ReMM score v0.4 on both genome builds, as well as average values (avg) with standard deviation in parentheses across 100 training runs. AUPRC and AUROC are computed via 10-fold cytoband cross-validation.

**Supplementary Table S4**. ReMM score correlation across genome builds—Pearson and Spearman correlation of ReMM scores between genome builds of 3 genic regions (DLK1, HBB, PRDM9) and 120,000 random positions (120 K). For 120 K, only variants with a successful coordinate liftOver from GRCh38 to GRCh37 and located on major human chromosomes are used (*n*= 110,751).

**Supplementary Table S5**. Feature value correlations across genome builds for regions and variants—average Pearson and Spearman correlation of the 26 features used for each genome build in three genic regions (DLK1, HBB, PRDM9) and 120,000 random positions (120 K). For 120 K, only variants with a successful coordinate liftOver from GRCh38 to GRCh37 and located on major human chromosomes are used (*n*= 110,751).

**Supplementary Table S6**. ReMM performance dependent on missing values—average area under the precision recall curve (AUPRC) and area under the receiver operating characteristic curve (AUROC) values with standard deviation in parentheses from 100 model training runs using zero as missing values or the default values listed in Supplementary Table S2. AUPRC and AUROC are computed via 10-fold cytoband cross-validation.

**Supplementary Table S7**. Feature importance—average feature importance (Gini index) over 100 random forest partitions of the hyperSMURF models of ReMM v0.4.hg19 and v0.4.hg38. Gini index values were derived with the Ranger package after training on all training data. The standard deviation (std), the minimum (min), and the maximum (max) values across the 100 partitions are shown. The rank indicates the importance rank by average Gini index.

**Supplementary Fig. S1**. ROC and PR curve of ReMM, CADD, ExPecto, and Sei for intersection of variants, scored by all tools—PR curves (A) and ROC curves (B) of ReMM v0.4.hg38 (10-fold cytoband cross-validation scores) as well as CADD v1.6, ExPecto, and Sei on the subset of the GRCh38 training data scored by all tools. In total, 406 pathogenic and 7,299,993 proxy-benign variants were scored. Area under the curve is shown in parentheses. ExPecto absmax is the maximum absolute value over all ExPecto outputs and ExPecto absmean the mean absolute value, respectively.

## Abbreviations

AUPRC: area under the precision recall curve; AUROC: area under the receiver operating characteristic curves; API: Application Programming Interface; BED: Browser Extensible Data format; CADD: Combined Annotation-Dependent Depletion; CV: cross-validation; ENCODE: encyclopedia of DNA elements; FANTOM: functional annotation of the mammalian genome; FP: false-positive; GC: guanine and cytosine nucleotides in a sequence; GRCh: Genome Reference Consortium for Human; NCBI: The National Center for Biotechnology Information; PR: precision-recall curve; ReMM: Regulatory Mendelian Mutation; REST: representation state transfer; RF: random forest; RRID: Research Resource Identification Initiative ID; ROC: receiver operating characteristic; SMOTE: Synthetic Minority Over-sampling Technique; SNV: single-nucleotide variant; TP: true-positive; UCSC: University of California, Santa Cruz; VCF: variant call format.
