# Supplementary material for: The Regulatory Mendelian Mutation score for GRCh38
Source: Gigascience. 2023 Apr 21;12:giad024. doi: 10.1093/gigascience/giad024 (PMC10120424; doi:10.1093/gigascience/giad024)
Supplement: giad024_GIGA-D-22-00232_Original_Submission [file giad024_giga-d-22-00232_original_submission.pdf]

GigaScience

The Regulatory Mendelian Mutation score for GRCh38

--Manuscript Draft--

**Manuscript Number:**	GIGA-D-22-00232
**Full Title:**	The Regulatory Mendelian Mutation score for GRCh38
**Article Type:**	Technical Note
**Funding Information:**	Helmholtz Einstein International Berlin Research School in Data Science — Ms. Lusiné Nazaretyan
**Abstract:**	Background<br>Various genome sequencing efforts for individuals with rare Mendelian disease have increased the research focus on the non-coding genome and the clinical need for methods that prioritize potentially disease causal non-coding variants. Some methods and annotations are not available for the current human genome build (GRCh38), for which the adoption in databases, software and pipelines was slow.<br>Results<br>Here, we present an updated version of the Regulatory Mendelian Mutation (ReMM) score, re-trained on features and variants derived from the GRCh38 genome build. Like its GRCh37 version, it achieves good performance on its highly imbalanced data. To improve accessibility and provide users with a toolbox to score their variant files and lookup scores in the genome, we developed a website and API for easy score lookup.<br>Conclusions<br>Scores of the GRCh38 genome build are highly correlated to the prior release with a performance increase due to the better coverage of features. For prioritization of non-coding mutations in imbalanced datasets, the ReMM score performed much better than other variation scores. Pre-scored whole genome files of GRCh37 and GRCh38 genome builds are available on Zenodo https://doi.org/10.5281/zenodo.6576087. The website, UCSC genome browser tracks, and an API are available at https://remm.bihealth.org.
**Corresponding Author:**	Max Schubach<br>Berlin Institute of Health at Charité - Universitätsmedizin Berlin<br>Berlin, GERMANY
**Corresponding Author Secondary Information:**	
**Corresponding Author's Institution:**	Berlin Institute of Health at Charité - Universitätsmedizin Berlin
**Corresponding Author's Secondary Institution:**	
**First Author:**	Max Schubach
**First Author Secondary Information:**	
**Order of Authors:**	Max Schubach
	Lusiné Nazaretyan
	Martin Kircher
**Order of Authors Secondary Information:**	
**Additional Information:**	
**Question**	**Response**
Are you submitting this manuscript to a special series or article collection?	No
**Experimental design and statistics**	Yes

Powered by Editorial Manager® and ProduXion Manager® from Aries Systems Corporation

Full details of the experimental design and statistical methods used should be given in the Methods section, as detailed in our Minimum Standards Reporting Checklist. Information essential to interpreting the data presented should be made available in the figure legends.<br><br>Have you included all the information requested in your manuscript?	
**Resources**<br><br>A description of all resources used, including antibodies, cell lines, animals and software tools, with enough information to allow them to be uniquely identified, should be included in the Methods section. Authors are strongly encouraged to cite Research Resource Identifiers (RRIDs) for antibodies, model organisms and tools, where possible.<br><br>Have you included the information requested as detailed in our Minimum Standards Reporting Checklist?	Yes
**Availability of data and materials**<br><br>All datasets and code on which the conclusions of the paper rely must be either included in your submission or deposited in publicly available repositories (where available and ethically appropriate), referencing such data using a unique identifier in the references and in the "Availability of Data and Materials" section of your manuscript.<br><br>Have you have met the above requirement as detailed in our Minimum Standards Reporting Checklist?	Yes

Manuscript Click here to access/download;Manuscript;ReMM.docx

The Regulatory Mendelian Mutation score for GRCh38

Max Schubach[1], Lusiné Nazaretyan[1], Martin Kircher[1,2]

[1] Berlin Institute of Health at Charité – Universitätsmedizin Berlin, Chariteplatz 1, Berlin, Germany
[2] Institute of Human Genetics, University Medical Center Schleswig-Holstein, University of Lübeck, Ratzeburger Allee 160, Lübeck, Germany

E-MAIL ADDRESSES:

Max Schubach: max.schubach@bih-charite.de
Lusiné Nazaretyan: lusine.nazaretyan@bih-charite.de
Martin Kircher: martin.kircher@bih-charite.de

ABSTRACT

Background

Various genome sequencing efforts for individuals with rare Mendelian disease have increased the research focus on the non-coding genome and the clinical need for methods that prioritize potentially disease causal non-coding variants. Some methods and annotations are not available for the current human genome build (GRCh38), for which the adoption in databases, software and pipelines was slow.

Results

Here, we present an updated version of the Regulatory Mendelian Mutation (ReMM) score, re-trained on features and variants derived from the GRCh38 genome build. Like its GRCh37 version, it achieves good performance on its highly imbalanced data. To improve accessibility and provide users with a toolbox to score their variant files and lookup scores in the genome, we developed a website and API for easy score lookup.

Conclusions

Scores of the GRCh38 genome build are highly correlated to the prior release with a performance increase due to the better coverage of features. For prioritization of non-coding mutations in imbalanced datasets, the ReMM score performed much better than other variation scores. Pre-scored whole genome files of GRCh37 and GRCh38 genome builds are available on Zenodo https://doi.org/10.5281/zenodo.6576087. The website, UCSC genome browser tracks, and an API are available at https://remm.bihealth.org.

KEYWORDS

variant prediction, machine learning, web service, mendelian disease, non-coding score, rare variant analysis, imbalanced data

FINDINGS

Introduction

The Regulatory Mendelian Mutation (ReMM) score predicts the potential pathogenicity of non-coding variants [1]. It is specifically designed for highly imbalanced datasets with an excess of neutral variants, which naturally occurs in whole genome sequencing of probands with Mendelian disorders because only a small number of variants are expected to be causal among thousands of observed variants. The original score was constructed on the human reference genome build GRCh37/hg19. Nowadays the standard for sequencing projects in clinic and research is the updated reference genome GRCh38/hg38. It contains new sequences at nearly 100 assembly gaps and reduces unresolved bases at about 3% of the genome [2]. Often coordinate liftovers are performed between builds but they are limited to well characterized regions in both genome builds and may be insensitive to changes in the exact sequence. In addition to the advantages of an updated reference genome, new annotations may primarily support GRCh38. This establishes a need for an update of the ReMM score and we present a version developed particularly for GRCh38. Further, we update the ReMM score for GRCh37 by including feature updates and improving its handling of missing values. We show that the score has superior performance on imbalanced datasets compared to competing approaches and the most frequently used scores in the field. Finally, we provide a webserver and API for scoring VCF files, single variant lookups or range lookups.

Methods

The ReMM score is based on an imbalance-aware machine learning algorithm, hyperSMURF [3], trained from known pathogenic non-coding variants of Mendelian disorders and a set of putatively benign variants. As pathogenic set, we use 406 hand-curated variants already used in the prior ReMM version [1], reciprocally lifted to GRCh38 using UCSC liftOver (v377) [4] and validated for identical allelic sequences. The proxy-benign set includes around 14 million of human-lineage-derived sequence alterations [5], which we filtered to non-coding sequence using Jannovar v0.36 [6] and RefSeq [7]. Restricting variants to non-coding only removes a small proportion of variants and the high imbalance with the pathogenic variant set is similar on both genome builds (14.8M and 13.9M negatives for GRCh37 and GRCh38, respectively). Therefore, we kept parameters for hyperSMURF model training as determined in Smedley et al. 2016 (Supplementary Table 1). Because of the large and computationally expensive dataset, we replaced hyperSMURF with the updated parSMURF implementation [8].

Genomic data is confounded by local correlation of annotations, i.e. gnomically proximal variants tend be more similar in their annotation results than random variants. Further, known pathogenic variants are not distributed evenly across the genome (e.g., due to selection bias), but rather cluster around certain well-studied genes and share certain molecular function properties due to shared identification or validation steps. When not accounted for, learners might infer superior hold-out performance because of genomic proximity of variants. To handle the local correlation structure, we apply cytogenic band-aware cross-validation using ten folds [1].

Twenty-six selected features (see Supplementary Table 2) capture functional constraint and sequence functions (sequence composition, epigenetics, conservation, population variance and regulatory regions) of the genetic variants. The feature set was kept close to the original feature set of ReMM, but some were not available from the original databases or were updated. Some features have a high proportion of missing values and the initial version of ReMM imputed all of them with zero. In genomics, a missing value often indicates an experimental signal that is too low to be measured, in line with this imputation. We have now identified some features (e.g., GC content or conservation scores) where the genome-wide average of the annotation is more appropriate and impute them differently in this version (see Supplementary Table 2). For missing p-values, we use the value 1.

Pre-scored, block-gzip compressed and indexed whole genome files [9] were generated to allow a fast scoring of variants as well as an easy integration into other software. Every genomic position was scored with a general ReMM model trained on all data (v0.4.hg19 and v0.4.hg38, respectively). To guarantee unbiased score usage, e.g., for performance benchmarks with other tools, we replaced the score of variants in the training set with cross-validated scores. The training and scoring pipeline is implemented in snakemake, a workflow management system for reproducible and scalable analysis [10].

Pre-scored GRCh38 whole genome files of CADD [5] version 1.6 were used to retrieve CADD raw scores on the ReMM training data for performance comparison. ExPecto [11] and Sei [12] scores were computed using VCF files as described on their source code repositories ([13], [14]). For ExPecto the UCSC hg19 fasta reference file was replaced with hg38 to retrieve scores on the new genome build. Sei was run with the --hg38 option, respectively. For ExPecto the absolute mean and absolute maximum over all 218 outputs is used as final score. Comparison was done against the cross-validated ReMM v0.4.hg38 scores.

Results

Performance of ReMM on GRCh38

After 100 training cycles using different random seeds and ten-fold cytoband cross validation, we achieve an excellent performance with an average area under the precision recall curve (AUPRC) of 0.613±0.005 (Supplementary Table 3). We randomly picked one model for the final scoring with an AUPRC of 0.610 (Figure 1a, receiver operating characteristic (ROC) performance available in Figure 1b).

Rather than using ReMM scores for ranking, some users need to specify score thresholds for classifying into pathogenic and benign variants. Using a cutoff of 0.5 yields a good result in terms of retrieving known pathogenic non-coding variants (i.e., recall or True Positive rate, TP), but the number of negatives might be extremely large. For ReMM v0.4.hg38, recall is 92% (375 out of 406) at a cutoff of 0.5 (Figure 1c), but precision is close to zero with lots of false positives (FP) (86,507 out of 13,911,061; FP rate=0.006). The F1-score (harmonic mean of recall and precision) is highest at 0.963, resulting in a TP rate of 0.554 and a FP rate of 5.3e-6. Using the F2-score, we can give more weight to recall. Here, the optimal cutoff is 0.914, resulting in a TP rate of 0.702 and a FP rate of 2.3e-5. Analogous to NCBI ClinVar [15] pathogenic and likely pathogenic categories, we suggest to use a ReMM score above the F1 threshold as weak computational evidence for "pathogenic" and a score above the F2 threshold and below the F1 threshold for "likely pathogenic". For ReMM v0.4.hg19, these thresholds are 0.961 and 0.924 (Figure 1d), respectively.

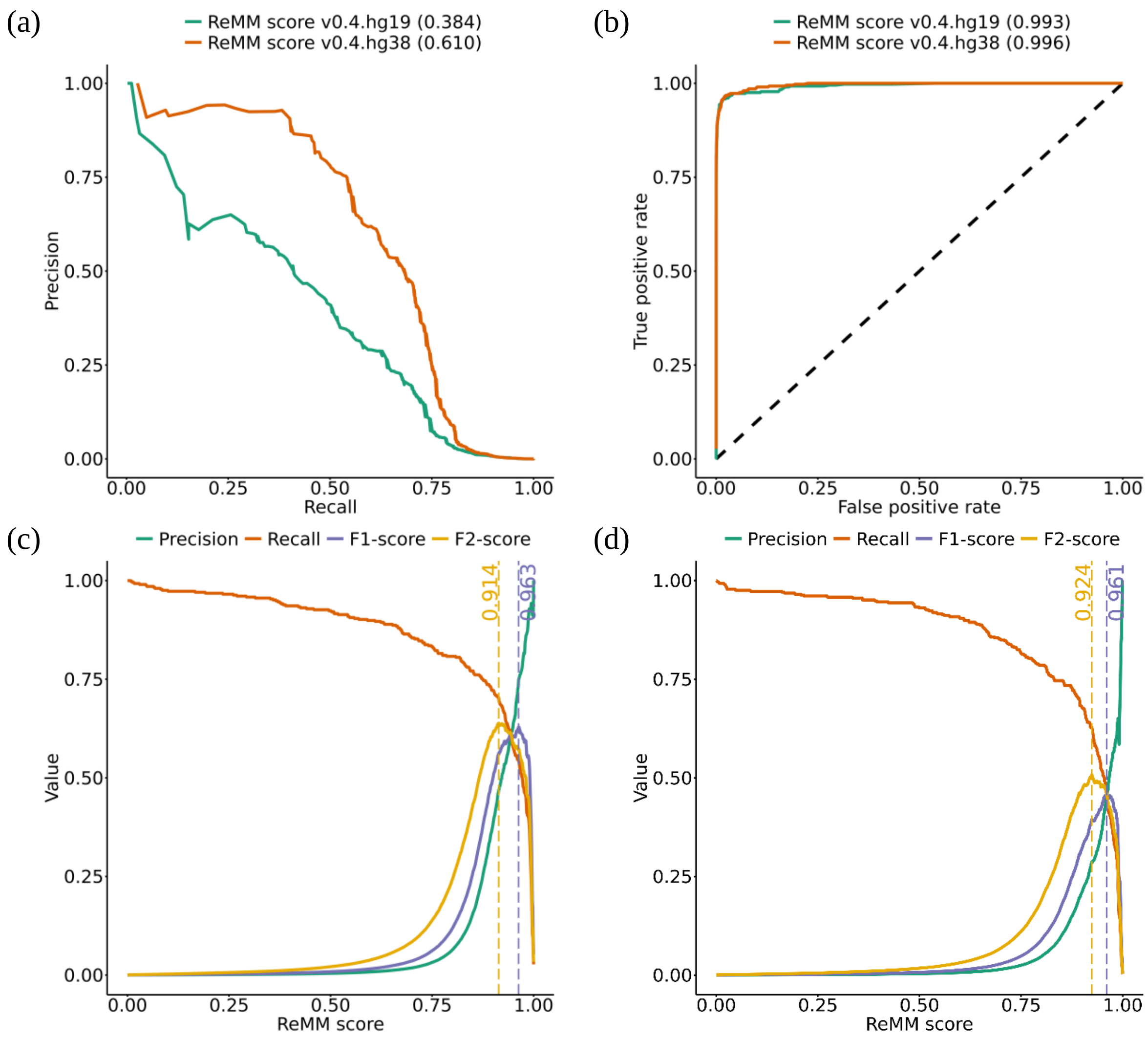

Figure 1: Precision, Recall, ROC, F1-, and F2-score curves – Performance metrics of ReMM v0.4.hg19 and v0.4.hg38 generated via ten-fold cytoband cross validation. Precision-Recall curves **(a)**, receiver operating characteristic (ROC) curves **(b)**, and precision, recall F1-score and F2-score (y-axis) over different ReMM score thresholds (x-axis) for v0.4.hg38 **(c)** and v0.4.hg19 **(d)**. Vertical lines denote the ReMM score with the maximum F1-score (yellow) and the maximum F2-score (purple). Area under the curve is shown in parentheses.

Correlation of scores and features

To compare both genome builds, we correlate ReMM scores from three genomic regions (genic content and not overlapping with assembly gap changes) and 120K randomly sampled positions and find that scores are highly correlated between versions (Supplementary Table 4). We also used these regions and sites to explore the average feature correlation (Supplementary Table 5). Further, we compare feature correlations between the genome builds directly on the training data (Figure 2). As expected from the high sequence similarity between reference sequence versions, we see the highest correlation for sequence features, like GC content. Further, population variance features correlate well, with reduced correlation for the rare variant feature. This is likely due to spurious calls highly depending on the caller and the quality of the reference genome. We see the lowest correlation on sparse Fantom5 regulatory element annotation data .

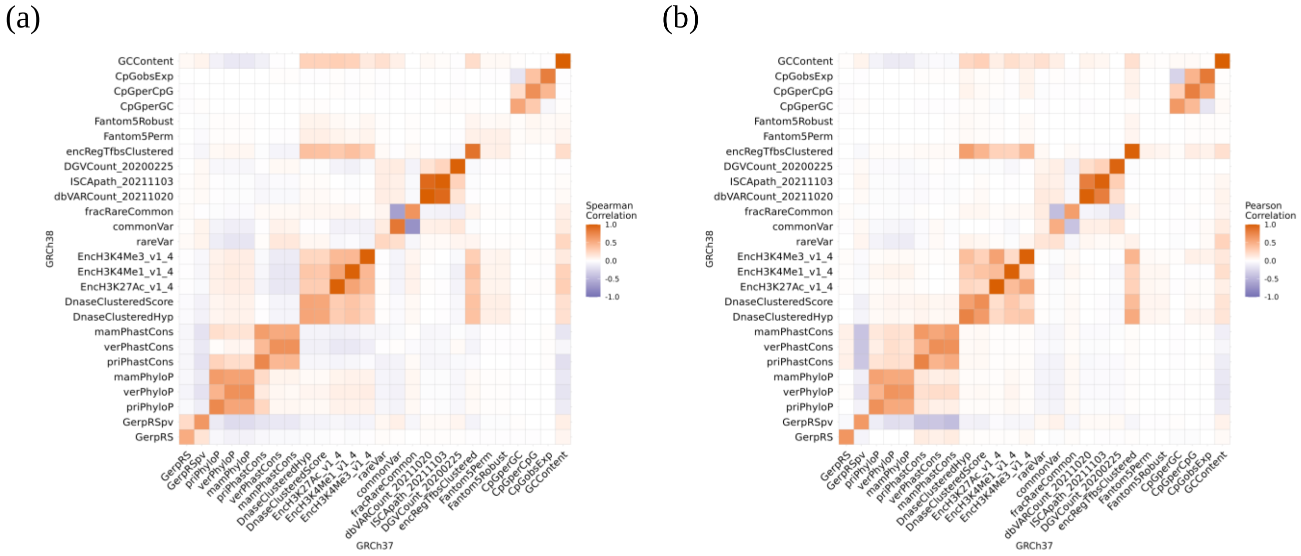

Figure 2: Correlation of feature values across genome builds – Feature correlation between features of the GRCh37 (x-axis) and the GRCh38 (y-axis) genome builds. The left heat map **(a)** shows Spearman correlation, and the right **(b)** shows Pearson correlation. Both plots show unexpectedly low correlations for some features along the diagonal. For example, the histone modification features (ENCODE) are lowly correlated as well as the enhancer features (FANTOM).

Imputing missing values

In previous ReMM versions, we used zero for missing values globally and trusted in the non-linearity of decision trees. Now, we use the average value of all defined positions for sequence and conservation features and one for p-values (see Supplementary Table 2). With the new approach, we see that the average AUPRC increases slightly (0.005 for v0.4.hg19, 0.009 for v0.4.hg38, Supplementary Table 6).

Feature importance

From the underlying Ranger random forest (RF) models [16], we retrieve feature importance using the Gini index. We averaged values over all 100 RFs in the model (Supplementary Table 7). In general, mean feature importance scores indicate contributions of all 26 features. No single feature stands out and our broad feature categories are all represented with at least one highly ranked feature. We interpret this as evidence that features were carefully picked and biases avoided. Epigenetic features increased in importance for the GRCh38 model (average rank 16 vs 19), maybe due to better mapping and processing of the underlying data. Fantom5 features are probably too sparse to receive high importance but might be relevant for some variants. Between genome builds, feature importance values are similar, and no significant changes are detected (p-value 0.565, two-sided ranksum test). The replaced encRegTfbsClustered feature achieves a similar average Gini index (rank 6 on v0.4.hg19) as the previous numTFBSConserved feature (rank 4, data not shown).

Comparison to other scores

A number of different tools for scoring pathogenicity of non-coding variants exist [17]. However, a majority of tools are still based on the GRCh37 genome build which comes with the previously discussed drawbacks when scores are lifted to a new genome build. To our knowledge, CADD, one of the most popular whole genome scores, seems to be the only tool directly trained on training data and features derived from GRCh38. Sequence based tools, like ExPecto [11] or Sei [12] are trained on the previous genome release but liftover can be avoided because predictions are solely based on sequence and the sequence around variants from GRCh38 can be used directly. We compared performance of ReMM with CADD v1.6 GRCh38, Sei, and ExPecto on the GRCh38 imbalanced training data. ExPecto annotated only 7,299,993 out of the 14 million negative variants, maybe due to missing transcripts close by. Therefore, performance might be overestimated. Area under the ROC curve above 0.8 for all tools (Figure 3b) but the area under the precision recall (PR) curve of ReMM substantially outperforms other methods (Figure 3a). It is worth noting that

in the context of extremely unbalanced data, the area under the PR curve is more informative than the area under the ROC curve [18].

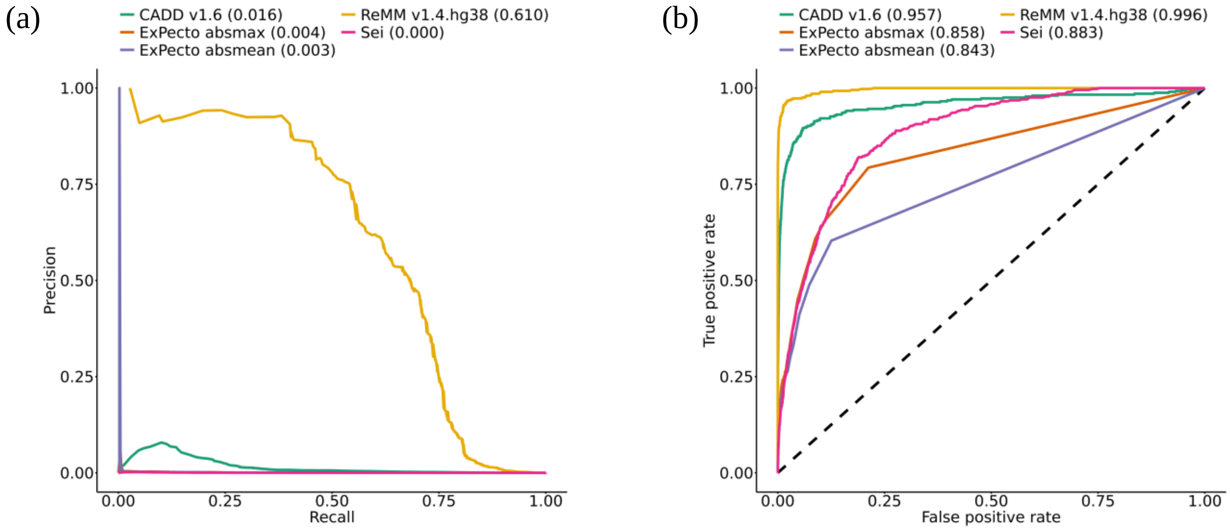

Figure 3: ROC and PR curve of ReMM, CADD, ExPecto, and Sei – Precision-Recall (PR) curves **(a)** and receiver operating characteristic (ROC) curves **(b)** of ReMM v0.4.hg38 (ten-fold cytoband cross validation scores) as well as CADD v1.6, v1.6, ExPecto, and Sei on the GRCh38 training data. Area under the curve is shown in parentheses. ExPecto absmax is the maximum absolute value over all ExPecto outputs and ExPecto absmean the mean absolute value, respectively.

Conclusion

The ReMM v0.4 score is a fully retrained non-coding score available for both the GRCh37 and GRCh38 genome builds. This fills the high need of supporting variant prioritization on the GRCh38 genome release, which is the *de facto* standard in research and routine diagnostics. Scores over the GRCh38 genome are highly correlated to the prior release with a performance increase due to the better coverage of features. On imbalanced data (commonly observed in whole genome sequencing of individuals affected with Mendelian disease), ReMM scores outperform other non-coding effect scores. In addition, we established a reproducible and scalable framework for integration of new features or new training data for further development of ReMM. The pre-scored whole genome files, UCSC genome browser annotation tracks [19] and a website provide fast access and easy usage of the ReMM score for researchers in all areas. With this release, tools like Genomiser [1] can now be run on the latest genome build, a highly demanded feature from the community.

AVAILABILITY OF SUPPORTING SOURCE CODE AND REQUIREMENTS

Project name: ReMM score

Project home page: https://remm.bihealth.org

Operating system(s): Platform independent (website), Linux (workflow)

Programming language: Python, Java, C++, Bash

Other requirements: browser (website); conda, snakemake, parSMURF (workflow)

License: MIT License

DATA AVAILABILITY

We precomputed ReMM scores for all sequence-resolved positions in the genome (GRCh37 and GRCh38 builds) and provide them on Zenodo (https://doi.org/10.5281/zenodo.6576087) or on the ReMM website https://remm.bihealth.org, where we enable fast and easy scoring of variants. Variants can be uploaded via a VCF file

[20], or scores directly displayed with a single site or genomic range variant lookup. Usage of UCSC genome browser tracks [19] of ReMM scores is described on the same websiteIn addition, we provide a REST-API that allows tools and scripts to retrieve ReMM scores directly. Scoring on the website is available for both genome builds and all major ReMM versions. The snakemake workflow to generate features, train scores and generate whole genome files is available on GitHub (https://github.com/kircherlab/ReMM, [21]).

DECLARATIONS

List of abbreviations

AUPRC: area under the precision recall curve
API: Application Programming Interface
CADD: Combined Annotation-Dependent Depletion
ENCODE: Encyclopedia of DNA elements
FANTOM: Functional annotation of the mammalian genome
GC: Guanine and Cytosine nucleotides in a sequence
GRCh: Genome Reference Consortium for Human
PR: precision recall curve
ReMM: Regulatory Mendelian Mutation
REST: Representation state transfer
RF: random forest
ROC: receiver operating characteristic
UCSC: University of California Santa Cruz
VCF: Variant Call Format

Ethics approval and consent to participate

Not applicable.

Consent for publication

Not applicable.

Competing interests

The authors declare that they have no competing interests.

Funding

Lusiné Nazaretyan is funded by the Helmholtz Einstein International Berlin Research School in Data Science (HEIBRiDS).

Authors' contributions

All authors designed the study. MS and LN prepared and analyzed the data. MS and LN wrote the software. All authors wrote the manuscript. All authors read and approved the submitted manuscript.

Acknowledgements

We thank current and previous members of the Kircher laboratory for helpful discussions and suggestions. Computation has been performed on the HPC for Research cluster of the Berlin Institute of Health at Charité – Universitätsklinikum Berlin.

REFERENCES

[1] D. Smedley *et al.*, “A Whole-Genome Analysis Framework for Effective Identification of Pathogenic Regulatory Variants in Mendelian Disease,” *Am. J. Hum. Genet.*, vol. 99, no. 3, pp. 595–606, 2016, doi: https://doi.org/10.1016/j.ajhg.2016.07.005.

[2] Y. Guo, Y. Dai, H. Yu, S. Zhao, D. C. Samuels, and Y. Shyr, “Improvements and impacts of GRCh38 human reference on high throughput sequencing data analysis,” *Genomics*, vol. 109, no. 2, pp. 83–90, März 2017, doi: 10.1016/j.ygeno.2017.01.005.

[3] M. Schubach, M. Re, P. N. Robinson, and G. Valentini, “Imbalance-Aware Machine Learning for Predicting Rare and Common Disease-Associated Non-Coding Variants,” *Sci. Rep.*, vol. 7, no. 1, p. 2959, Jun. 2017, doi: 10.1038/s41598-017-03011-5.

[4] B. T. Lee *et al.*, “The UCSC Genome Browser database: 2022 update,” *Nucleic Acids Res.*, vol. 50, no. D1, pp. D1115–D1122, Jan. 2022, doi: 10.1093/nar/gkab959.

[5] P. Rentzsch, D. Witten, G. M. Cooper, J. Shendure, and M. Kircher, “CADD: predicting the deleteriousness of variants throughout the human genome,” *Nucleic Acids Res.*, vol. 47, no. D1, pp. D886–D894, Jan. 2019, doi: 10.1093/nar/gky1016.

[6] M. Jäger, K. Wang, S. Bauer, D. Smedley, P. Krawitz, and P. N. Robinson, “Jannovar: A Java Library for Exome Annotation,” *Hum. Mutat.*, vol. 35, no. 5, pp. 548–555, 2014, doi: 10.1002/humu.22531.

[7] N. A. O’Leary *et al.*, “Reference sequence (RefSeq) database at NCBI: current status, taxonomic expansion, and functional annotation,” *Nucleic Acids Res.*, vol. 44, no. D1, pp. D733–D745, Jan. 2016, doi: 10.1093/nar/gkv1189.

[8] A. Petrini *et al.*, “parSMURF, a high-performance computing tool for the genome-wide detection of pathogenic variants,” *GigaScience*, vol. 9, no. 5, 2020, doi: 10.1093/gigascience/giaa052.

[9] H. Li, “Tabix: fast retrieval of sequence features from generic TAB-delimited files,” *Bioinformatics*, vol. 27, no. 5, pp. 718–719, März 2011, doi: 10.1093/bioinformatics/btq671.

[10] F. Mölder *et al.*, “Sustainable data analysis with Snakemake.” F1000Research, Jan. 18, 2021. doi: 10.12688/f1000research.29032.1.

[11] J. Zhou, C. L. Theesfeld, K. Yao, K. M. Chen, A. K. Wong, and O. G. Troyanskaya, “Deep learning sequence-based ab initio prediction of variant effects on expression and disease risk,” *Nat. Genet.*, vol. 50, no. 8, Art. no. 8, Aug. 2018, doi: 10.1038/s41588-018-0160-6.

[12] K. M. Chen, A. K. Wong, O. G. Troyanskaya, and J. Zhou, “A sequence-based global map of regulatory activity for deciphering human genetics,” *Nat. Genet.*, pp. 1–10, Jul. 2022, doi: 10.1038/s41588-022-01102-2.

[13] *ExPecto*. Troyanskaya Laboratory, 2022. Accessed: Jul. 15, 2022. [Online]. Available: https://github.com/FunctionLab/ExPecto

[14] *Sei framework*. Troyanskaya Laboratory, 2022. Accessed: Jul. 15, 2022. [Online]. Available: https://github.com/FunctionLab/sei-framework

[15] M. J. Landrum *et al.*, “ClinVar: improving access to variant interpretations and supporting evidence,” *Nucleic Acids Res.*, vol. 46, no. D1, pp. D1062–D1067, Jan. 2018, doi: 10.1093/nar/gkx1153.

[16] M. N. Wright and A. Ziegler, “ranger: A Fast Implementation of Random Forests for High Dimensional Data in C++ and R,” *J. Stat. Softw.*, vol. 77, pp. 1–17, Mar. 2017, doi: 10.18637/jss.v077.i01.

[17] Z. Wang *et al.*, “Performance comparison of computational methods for the prediction of the function and pathogenicity of non-coding variants,” *Genomics Proteomics Bioinformatics*, Mar. 2022, doi: 10.1016/j.gpb.2022.02.002.

[18] T. Saito and M. Rehmsmeier, “The Precision-Recall Plot Is More Informative than the ROC Plot When Evaluating Binary Classifiers on Imbalanced Datasets,” *PLOS ONE*, vol. 10, no. 3, p. e0118432, Mar. 2015, doi: 10.1371/journal.pone.0118432.

[19] W. J. Kent *et al.*, “The Human Genome Browser at UCSC,” *Genome Res.*, vol. 12, no. 6, pp. 996–1006, Jan. 2002, doi: 10.1101/gr.229102.

[20] P. Danecek *et al.*, “The variant call format and VCFtools,” *Bioinformatics*, vol. 27, no. 15, pp. 2156–2158, Aug. 2011, doi: 10.1093/bioinformatics/btr330.

[21] *ReMM*. KircherLab. Accessed: Jul. 15, 2022. [Online]. Available: https://github.com/kircherlab/ReMM

Click here to access/download
Supplementary Material
ReMM_Supplement.docx
