# Supplementary material for: The Regulatory Mendelian Mutation score for GRCh38
Source: Gigascience. 2023 Apr 21;12:giad024. doi: 10.1093/gigascience/giad024 (PMC10120424; doi:10.1093/gigascience/giad024)
Supplement: giad024_GIGA-D-22-00232_Revision_1 [file giad024_giga-d-22-00232_revision_1.pdf]

GigaScience

The Regulatory Mendelian Mutation score for GRCh38

--Manuscript Draft--

**Manuscript Number:**	GIGA-D-22-00232R1
**Full Title:**	The Regulatory Mendelian Mutation score for GRCh38
**Article Type:**	Technical Note
**Funding Information:**	Helmholtz Einstein International Berlin Research School in Data Science — Ms. Lusiné Nazaretyan
**Abstract:**	Background<br>Genome sequencing efforts for individuals with rare Mendelian disease have increased the research focus on the non-coding genome and the clinical need for methods that prioritize potentially disease causal non-coding variants. Some tools for assessment of variant pathogenicity as well as annotations are not available for the current human genome build (GRCh38), for which the adoption in databases, software and pipelines was slow.<br>Results<br>Here, we present an updated version of the Regulatory Mendelian Mutation (ReMM) score, re-trained on features and variants derived from the GRCh38 genome build. Like its GRCh37 version, it achieves good performance on its highly imbalanced data. To improve accessibility and provide users with a toolbox to score their variant files and lookup scores in the genome, we developed a website and API for easy score lookup.<br>Conclusions<br>Scores of the GRCh38 genome build are highly correlated to the prior release with a performance increase due to the better coverage of features. For prioritization of non-coding mutations in imbalanced datasets, the ReMM score performed much better than other variation scores. Pre-scored whole genome files of GRCh37 and GRCh38 genome builds are available on Zenodo https://doi.org/10.5281/zenodo.6576087. The website, UCSC genome browser tracks, and an API are available at https://remm.bihealth.org.
**Corresponding Author:**	Max Schubach<br>Berlin Institute of Health at Charité - Universitätsmedizin Berlin<br>Berlin, GERMANY
**Corresponding Author Secondary Information:**	
**Corresponding Author's Institution:**	Berlin Institute of Health at Charité - Universitätsmedizin Berlin
**Corresponding Author's Secondary Institution:**	
**First Author:**	Max Schubach
**First Author Secondary Information:**	
**Order of Authors:**	Max Schubach
	Lusiné Nazaretyan
	Martin Kircher
**Order of Authors Secondary Information:**	
**Response to Reviewers:**	We thank all reviewers and editor for their kind and constructive feedback on our manuscript. We addressed the additional comments brought up by the reviewers as well as the further requirements mentioned by the editor. We address each point in this response individually. Sections altered in the manuscript are highlighted in this document.<br><br>===================================<br>Comments and tasks assigned by the editor<br>===================================

Powered by Editorial Manager® and ProduXion Manager® from Aries Systems Corporation

* (editor) please register any new software application in the bio.tools and SciCrunch.org databases to receive RRID (Research Resource Identification Initiative ID) and biotoolsID identifiers, and include these in your manuscript. Computational workflows should be registered in workflowhub.eu and the DOIs cited in the relevant places in the manuscript. These will facilitate tracking, reproducibility and re-use of your tool.

(response) We have registered ReMM and provide its identifier (biotools:remm_score; RRID: SCR_023095; https://doi.org/ 10.48546/workflowhub.workflow.414.1) in the manuscript.

* (editor) Please include a point-by-point within the 'Response to Reviewers' box in the submission system. Please ensure you describe additional experiments that were carried out and include a detailed rebuttal of any criticisms or requested revisions that you disagreed with.

(response) We followed the editors' instructions, and this document provides the requested ‘Response to Reviewers’ document.

* (editor) Please also ensure that your revised manuscript conforms to the journal style, which can be found in the Instructions for Authors on the journal homepage. If the data and code has been modified in the revision process please be sure to update the public versions of this too.

(response) We made all required updated and we checked the author’s instructions again and believe that our manuscript matches all requirements.

=================
Reviewer comments
=================

Reviewer #1
----
1. (reviewer #1) In the abstract "Some methods and annotations are not available for the current human genome build (GRCh38), for which the adoption in databases, software and pipelines was slow." Not sure what the author is referring to by some methods, this could be a grammar problem.

(response) Thank you for this comment. We assume that the reviewer considers “methods” to be universal and independent of their exact implementation for a specific reference genome build, and that this causes some confusion. Here, we use “methods” as synonymous with “tools”. We modified the sentence to “Some tools for assessment of variant pathogenicity as well as annotations are not available for the current human genome build (GRCh38), for which the adoption in databases, software and pipelines was slow.” We hope this makes it clearer.

2. (reviewer #1) "Restricting variants to non-coding only removes a small proportion of variants", what is the proportion? Also, I don't understand the need to remove coding variants, shouldn't your model works also with coding variants?

(response) The ReMM score as originally proposed by Smedley D et al. (Am J Hum Genet. 2016 Sep 1;99(3):595-606. doi: 10.1016/j.ajhg.2016.07.005) is a score for non-coding sequence variation. We remove the coding variants in the benign set to match the pathogenic training set which contains only non-coding variants. There is considerably more training data for coding variants, their inclusion in training would overshadow the small number of described pathogenic non-coding variants for which we aim to train a dedicated model. We added the proportion of dropped variants in the manuscripts (1% for GRCh37 and 0.7% for GRCh38).

3. (reviewer #1) The method the author used is based on a previous publication. However, there is still the need to give the detail of the method in this manuscript. There is a lot of missing information. For example, what is the outcome, whether a position is deleterious? How is the probability for deleteriousness calculated?

(response) Thank you for the feedback. Indeed, we were very short in our methods, with details only available from the respective references. This makes it difficult to read for anyone not familiar with the previous work. Therefore, we extended the method section and provide more information to the reader.

hyperSMURF/parSMURF:

"The hyperSMURF algorithm applies a special sampling technique essential for the highly imbalanced data of human pathogenic variants [1], [5]. The minority class (for ReMM the pathogenic variants) is oversampled based on the Synthetic Minority Over-sampling Technique (SMOTE) that creates synthetic examples using k-nearest neighbors rather than over-sampling the data with replacement [11]. The majority class (proxy-benign set) is divided into n non-overlapping partitions, which then are subsampled according to a ratio parameter. The minority class is oversampled by factor 2 and the majority class is undersampled by factor 3 which leads to the ration of pathogenic vs. benign variants of 2-3 in a more balanced dataset with around 2,000 datapoints. However, each balanced dataset alone provides insufficient coverage of the large data space of the majority class. That is why hyperSMURF applies an ensemble method: it divides the dataset into 100 partitions each containing all oversampled pathogenic and one partition of downsampled proxy-benign variants. On each partition, a random forest [12] is trained and the final pathogenicity score is the average over the 100 predictions. It ranges from 0 (not pathogenic) to 1 (pathogenic) and gives the probability values of a variant to belong to the pathogenic training data. Thus, the higher the score, the more likely that a variant at that position is pathogenic. We used parSMURF as implementation of hyperSMURF, a fast and highly scalable model training tool based on random forest algorithms [10]."

Ten-fold cytogenic band-aware cross-validation:

"To handle the local correlation structure in the genome, we apply ten-fold cytogenic band-aware cross-validation (CV) [1]. This is a stratified CV approach where each cytoband of the genome is associated to one out of ten folds. Folds are assigned to have a similar number of pathogenic variants, cytobands without pathogenic variants are randomly assigned to a fold. Proxy-benign variants are considered in the folds of their associated bands. Thereby gnomically proximal (i.e., same cytoband) pathogenic and proxy-benign variants are considered together, making it more challenging for the learner to discriminate between the two groups. Ten separate models are trained on nine folds and validated on the tenth fold. Unbiased predictions of variants contained in the training set can be performed with the model that only used the variants in the validation fold, while other variants are reported as the average of the ten model predictions."

4. (reviewer #1) by a few specific variants. Thus, the overall Mendelian disease-related variants should be low. I am guessing that's why 406 hand-curated variants were used in the previous version of ReMM. If my assumption is correct, there shouldn't be a lot variants for Mendelian disease. How many variants are found to be positive in the entire genome?

(response) That is correct, the same pathogenic set was used for the training of the previous ReMM version. We also agree that we would not expect many Mendelian disease-related non-coding variants in the whole genome, however there is also not much empirical data to substantiate any specific claims. Using a mostly coding-based diagnostic approach, current studies identify disease-causing variants in up to 40% of large patient cohorts, leaving a substantial proportion of patients with a potential non-coding cause. As of Jan 2022, there were about 85,000 coding pathogenic/likely pathogenic variants reported in NCBI ClinVar for what accounts to about 1.5% of the genome. However, more than 366,000 variants were reported as variants of uncertain clinical significance for that same part of the genome. Large-scale efforts like TopMed/BRAVO or gnomAD cataloged about 800 million variants genome-wide, which is still a minority of all possible 9 billion single nucleotide changes to the human genome. Given all these considerations, it is unclear how many pathogenic variants ReMM should predict when applied to the whole genome.

To perform the analysis suggested by the reviewer, we counted all non-N positions in the human reference genome (GRCh38: 2,923,716,084, GRCh37: 2,862,362,542). We then determined those that are above the maximum F2 score (Figure 1 c and d). In total, the ReMM score predicts 25,734,725 pathogenic positions (0.9%) for GRCh37 and 37,888,567 positions (1.3%) for GRCh38. We note a considerable enrichment of

coding regions in the predicted pathogenic positions (34%, n=8,957,838 for GRCh37/GENCODE v19); 35%, n=13,540,527 for GRCh38/GENCODE v42). It is likely that this is due to higher conservation scores in coding regions, another reason why we excluded coding effect in training and recommend that specialized coding scores should be used in this case.

5. (reviewer #1) In the online application, the results are limited to 500, the rest cannot be seen or downloaded. I would be better to allow the user to download the entire results.

(response) This comment probably refers to the range lookup (https://remm.bihealth.org/range-lookup). Here, we limit the output to 500 because the result is directly returned to the user's web browser. This part of our website only allows the user to download or copy results printed on the page. Larger ranges will cause performance issues for the browser. In this case, provided whole genome wide scoring files (indexed for direct retrieval of results using htslib/tabix or similar tools) as well as the UCSC browser tracks are much more useful. To help the user to retrieve larger ranges of scores, we added a note to the website that states the range limit and points to the existing solutions when scores of larger regions are needed.

6. (reviewer #1) The authors performed comparison with other tools and generated ROC curve which is dependent on knowing the true positives. There is no description of the dataset that was used for the comparison. Did the authors make sure that the training variants is not used for the comparison?

(response) We added further score / tool comparisons (e.g., CADD, LINSIGHT, fathmm-MKL, NCBoost, Expecto, Sei, RegBase) on a validation set based on GRCh37 NCBI ClinVar variants, for which any overlap with the training data set has been excluded. New plots can be found in the manuscript as Figure 4a and b. We further added a section in the methods with the description of the ClinVar dataset. We copy the text here:
"Due to the very limited availability of non-coding scores on GRCh38, we compared ReMM on GRCh37 with multiple other scores and on a set of non-coding variants from NCBI ClinVar that do not overlap its training set. We only used variants where all scores were able to provide a prediction (869 pathogenic and 190,548 benign) and plotted PR and ROC curves (Figure 4a and Figure 4b). CADD v1.6 achieved the best performance in terms of AURPC (0.160) and area under the ROC curve (AUROC, 0.811) followed by the (liftover) GRCh38 version of ReMM (AUPRC=0.035, AUROC=0.694). Interestingly, CADD v1.3, a previous version that does not yet include features for intronic splice variants, has a much lower performance on the non-coding ClinVar dataset (AUPRC=0.012, AUROC=0.633). We therefore speculate, that the performance boost in CADD v1.6 is due to the presence of many splice variants in the non-coding ClinVar dataset and the inclusion of specific splice-scores, like SpliceAI [33] or MMSsplice [34], in recent CADD versions. All compared scores, excluding CADD v1.6, are not optimized for splicing effects. Further, ReMM's pathogenic training set does neither contain splice variants nor did we add specific splicing features with this update."

Reviewer #2
----
1. (reviewer #2) How to deal with missing value variants in test datasets when compare new ReMM with other tools, the author mentioned that ExPecto annotated only half of the million negative variants.

(response) Yes, ExPecto was only able to predict 7 million variants of our proxy benign set. For all performance metrices/curves, we used all data that a score was able to predict. Therefore, performance measurements of ExPecto were only done on half of the proxy benign set. We had already mentioned in the manuscript that this strategy might overestimate performance for scores with a lot of missing values. A second option would be using all not predictable variants as false classifications, which we considered an overly harsh approach. As a third option, comparison could be limited to (a potentially small intersection of) variants for which all tools are able to produce predictions. We now include a figure showing the comparison on the intersection of

Powered by Editorial Manager® and ProduXion Manager® from Aries Systems Corporation

variants in the supplement (Supplementary Figure 1). Order and general results are stable despite the difference in the number of variants scored.

2. (reviewer #2) Although the CADD used the same negative training dataset, it's not suitable to compare it in the ReMM training dataset. How those tools performance in the independent test datasets.

(response) We agree that training datasets and objectives of CADD and ReMM are very different, and that the training performance should not be compared. This was also not our intention. ReMM uses a manually curated pathogenic set and contrasts that with an evolutionary derived benign variant set. This happens to be part of the CADD training dataset, but CADD uses it in a surrogate training approach (with substantial mislabeling and low accuracy on its training data) where random variants are contrasted with variants that have seen many generations of purifying selection to derive a measure of deleteriousness. Our intention was to analyze whether CADD can identify the manually curated pathogenic variants that ReMM uses for its training. Focusing on precision-recall, it turns out that it does so better than some state-of-the-art sequence-based predictors. We agree that this analysis has its limitations, due to the overlap of the training datasets as the reviewer(s) point out, but with CADD's own training accuracy of only 62.2% (GRCh38-v1.6), it is also not as circular as one might think. Further, we are using the cross-validation-derived ReMM scores. However, to make an unbiased comparison we added a comparison on the NCBI ClinVar set of pathogenic and benign variants that were not present in any training set (see response reviewer #1, point 6).

3. (reviewer #2) The author presumes that new genome build will get better performance, is there some evidence can support this perspective, like the distribution of feature or training data in different genome build.

(response) The new genome build closed over 100 gaps and increased the number of resolved bases (e.g., 2,923,716,084 non-N positions in GRCh38 vs 2,862,362,542 non-N positions in GRCh37). This means that features based on sequence comparisons, like species conservation or chromatin assays, can be expected to be more accurate. For example, for the 26 features of our pathogenic variant set (n=406), the number of missing values reduced from 2,390 for GRCh37 to 2,154 for GRCh38 (proxy-benign from 140,548,778 down to 131,133,851). Similarly, on the >100k positions (i.e., positions with reciprocal liftover used in our correlation analysis between the genome builds), we see a reduction in missing values from 1,071,510 to 1,067,415. This allows the model a better distinction between the two class labels. We can also show this effect using a Wilcoxon Rank Sum (WRS) test between the two class labels for all individual features. While for GRCh38 all but one feature (DGVCount_20200225) shows a significant shift between the labels, for GRCh37 four features show a non-significant shift (Fantom5Perm, Fantom5Robust, DGVCount_20200225, rareVar).

4. (reviewer #2) Other existing similar tools can prioritization disease-causal noncoding variant, such as regBase-PAT, NCBoost, ncER, etc. can the authors compare new version of ReMM with these tools.

(response) We agree that there are other scores that can be used to prioritize non-coding variants. But unfortunately, all scores are limited to the GRCh37 genome release, limiting their use in new sequencing projects in clinic and research. Coordinate liftover can be performed between builds but has clear limitations when it comes to newly assembled regions and (depending on use) might be insensitive to changes in the exact sequence. This is one of the main motivations to build a new ReMM score for the GRCh38 release. To perform a sensible comparison, we decided to lift the GRCh38 score to GRCh37 and provide a comparison with several of the state-of-the-art non-coding scores (i.e., CADD, LINSIGHT, fathmm-MKL, NCBoost, Expecto, Sei, RegBase) on a ClinVar benchmark (Figure 4). We stress however, that the number of scores that can be applied to GRCh38 without liftover is very limited.

Reviewer #3

Powered by Editorial Manager® and ProduXion Manager® from Aries Systems Corporation

(reviewer #3) This is a valuable resource for the community of researchers and clinicians working on the interpretation of genetic variants in the human genome. The work appears to be thoughtfully done and appropriate assessments have been provided. The use of the random forest models to weigh the contributions of features was particularly noted for the insights it provided into how features contribute to prediction.

(response) Thank you for the kind review of our work.

(reviewer #3) My biggest concerns are stylistic, which falls outside the scientific quality of the work. I provide these comments for the authors to consider and do not expect that my stylistic preferences will be uniformly accepted. A fair amount of justification of the manuscript focuses on the value of having a release for version 38 of the human genome, pointing to the field as not having done so broadly. I think this is misguided, as by the time people are reading the manuscript such points will have lost relevance. I suggest a focus on the science be given, as there is no need to justify things based on where other resources have progressed in releasing their version 38 updates. Points below include language/text clarifications that can be assessed by the authors. Writing styles differ, so stylistic comments should be optional.

(response) Thank you for this insightful comment. We are a little torn here. We feel that our critique of a field that has very slowly adapted to GRCh38 and that keeps arguing with the small number of sequence changes as a reason to not adjust tools and pipelines is still very timely. Recent publications of the Telomere-to-Telomere (T2T) consortium have been very clear about the effects of missing genes, incorrect assemblies and artifacts of aligning to an incomplete reference. We believe that there might be some critical progress on the horizon. We also understand that our criticism will be dated quickly. However, each publication has its historic context in which it was written and ours has been written with some level of frustration over the adoption of GRCh38 in the community. Anyways, we thank the reviewer for that valuable and positive feedback on our work. We went through your minor points and incorporated your suggestions. Please see below for a detailed response.

1. (reviewer #3) The word "various" is vague and often shows up when people are too busy to provide an accurate statement. Starting the manuscript with it makes a bad impression on this reader. You do not have to change it, but I thought you might appreciate knowing this impression. You could delete it with no harm to the sentence. (Not to get carried away, but the next sentence starting with "some" heightens the impression of 'hand waving'.)

(response) Thank you! Yes, we appreciate your clarification here. We deleted “various” in the abstract and are not using it throughout the manuscript. We will consider its usage carefully in future manuscripts.

2. (reviewer #3) I think I understand ", we apply cytogenic band-aware cross-validation using ten folds" but I encourage the authors to provide clearer wording for this point.

(response) We revised the method section and added a paragraph explaining the cytogenic band-aware cross-validation as outlined above.

3. (reviewer #3) I would allow the reader to make their own judgement of performance. So please remove "excellent" from "we achieve an excellent performance"
The word “excellent” was removed from the manuscript.

4. (reviewer #3) "Rather than using ReMM scores for ranking, some users need to specify score thresholds" is confusing. I would change 'need to' to 'choose to'

(response) We changed “need to” to “choose to”.

5. (reviewer #3) "with lots of false positives" is a bit informal. I suggest "with a high false positive rate"

(response) We modified it as suggested.

Powered by Editorial Manager® and ProduXion Manager® from Aries Systems Corporation

	6 (reviewer #3) I am confused by "from three genomic regions (genic content and not overlapping with assembly gap changes) " as the brackets include two items, not three.<br><br>(response) We modified the section to include more details which were only available in the supplement. The new section reads as follows:<br>“To compare both genome builds, we correlate ReMM scores from three genomic regions without assembly gap changes (DLK1, HBB, and PRDM9 loci) and >100,000 randomly sampled autosomal positions with successful reciprocal liftover (Supplementary Table 4). Here, ReMM scores are highly correlated between versions (Spearman and Pearson correlation between 0.7 and 0.8, Supplementary Table 4). We also used these regions and sites to explore the average feature correlation and find those to be similar, but except for one region (PRDM9) to be lower (Spearman correlation of 0.7 and Pearson correlation between 0.6-0.8, Supplementary Table 5).”<br><br>7. (reviewer #3) "maybe due to better mapping" - "maybe" should be "may be"<br><br>(response) Thank you. We corrected it.<br><br>8. (reviewer #3) I think the language like "seems to be the only tool directly trained on training data and features derived from GRCh38." Is not particularly valuable long term. This is a useful contribution, but many tools are being updated to 38 and by the time this appears and is read, such statements decline in relevance. I would focus on providing this valuable resource, and not try to justify it based on a transient perception of where the field stands in updating versions.<br><br>(response) We agree, but there is considerable hesitation to update or to even develop new tools on the current reference genome. We rephrased to make clear that 9 years after the GRCh38 release, only one score got updated on GRCh38 and that no other score exists for a direct comparison on GRCh38:<br>“However, a majority of tools are still based on the GRCh37 genome build which comes with the previously discussed drawbacks when scores are lifted to a new genome build[3], [4], [31]. To our knowledge, CADD, one of the most popular whole genome scores, seems to be the only tool directly trained on training data and features derived from GRCh38. Nine years after the GRCh38 release, no other non-coding score is adapted to the new genome build. Also, more recent sequence-based tools, like ExPecto [16] or DeepSEA-Sei [17] are trained on the previous genome release.”<br><br>9. (reviewer #3) "It is worth noting that in the context of extremely unbalanced data…" - you do note it. So I would change the wording to "In the context of extremely unbalanced data…"<br><br>(response) Thank you. We corrected that.
**Additional Information:**	
**Question**	**Response**
Are you submitting this manuscript to a special series or article collection?	No
**Experimental design and statistics**<br><br>Full details of the experimental design and statistical methods used should be given in the Methods section, as detailed in our Minimum Standards Reporting Checklist. Information essential to interpreting the data presented should be made available in the figure legends.	Yes

Have you included all the information requested in your manuscript?	
**Resources**<br><br>A description of all resources used, including antibodies, cell lines, animals and software tools, with enough information to allow them to be uniquely identified, should be included in the Methods section. Authors are strongly encouraged to cite Research Resource Identifiers (RRIDs) for antibodies, model organisms and tools, where possible.<br><br>Have you included the information requested as detailed in our Minimum Standards Reporting Checklist?	Yes
**Availability of data and materials**<br><br>All datasets and code on which the conclusions of the paper rely must be either included in your submission or deposited in publicly available repositories (where available and ethically appropriate), referencing such data using a unique identifier in the references and in the “Availability of Data and Materials” section of your manuscript.<br><br>Have you have met the above requirement as detailed in our Minimum Standards Reporting Checklist?	Yes

Powered by Editorial Manager® and ProduXion Manager® from Aries Systems Corporation

Manuscript Click here to access/download;Manuscript;ReMM-March-22.docx

The Regulatory Mendelian Mutation score for GRCh38

Max Schubach[1], Lusiné Nazaretyan[1], Martin Kircher[1,2]

[1] Berlin Institute of Health at Charité – Universitätsmedizin Berlin, Charitéplatz 1, Berlin, Germany
[2] Institute of Human Genetics, University Medical Center Schleswig-Holstein, University of Lübeck, Ratzeburger Allee 160, Lübeck, Germany

E-MAIL ADDRESSES:

Max Schubach: max.schubach@bih-charite.de
Lusiné Nazaretyan: lusine.nazaretyan@bih-charite.de
Martin Kircher: martin.kircher@bih-charite.de

Max Schubach [0000-0002-2032-6679];
Lusiné Nazaretyan [0000-0001-5820-4143];
Martin Kircher [0000-0001-9278-5471]

ABSTRACT

Background

Genome sequencing efforts for individuals with rare Mendelian disease have increased the research focus on the non-coding genome and the clinical need for methods that prioritize potentially disease causal non-coding variants. Some tools for assessment of variant pathogenicity as well as annotations are not available for the current human genome build (GRCh38), for which the adoption in databases, software and pipelines was slow.

Results

Here, we present an updated version of the Regulatory Mendelian Mutation (ReMM) score, re-trained on features and variants derived from the GRCh38 genome build. Like its GRCh37 version, it achieves good performance on its highly imbalanced data. To improve accessibility and provide users with a toolbox to score their variant files and lookup scores in the genome, we developed a website and API for easy score lookup.

Conclusions

Scores of the GRCh38 genome build are highly correlated to the prior release with a performance increase due to the better coverage of features. For prioritization of non-coding mutations in imbalanced datasets, the ReMM score performed much better than other variation scores. Pre-scored whole genome files of GRCh37 and GRCh38 genome builds are cited in the paper and the website, UCSC genome browser tracks, and an API are available at https://remm.bihealth.org.

KEYWORDS

variant prediction, machine learning, web service, mendelian disease, non-coding score, rare variant analysis, imbalanced data

FINDINGS

Introduction

The Regulatory Mendelian Mutation (ReMM) score predicts the potential pathogenicity of non-coding variants [1]. It is specifically designed for highly imbalanced datasets with an excess of neutral variants, which naturally occurs in whole genome sequencing of probands with Mendelian disorders because only a small number of variants are expected to be causal among thousands of observed variants. The original score was constructed on the human reference genome build GRCh37/hg19. Nowadays the standard for sequencing projects in clinic and research is the updated reference genome GRCh38/hg38. It contains new sequences at nearly 100 assembly gaps and reduces unresolved bases at about 3% of the genome [2]. Often coordinate liftovers are performed between builds but they are limited to well characterized regions in both genome builds and may be insensitive to changes in the exact sequence. In addition to the advantages of an updated reference genome [3,4], new annotations may primarily support GRCh38. This

establishes a need for an update of the ReMM score and we present a version developed particularly for GRCh38. Further, we update the ReMM score for GRCh37 by including feature updates and improving its handling of missing values. We show that the score has superior performance on imbalanced datasets compared to competing approaches and the most frequently used scores in the field. Finally, we provide a webserver and API for scoring VCF files, single variant lookups or range lookups.

Methods

Training set labels and hyper-parameters

The ReMM score is based on an imbalance-aware machine learning algorithm, hyperSMURF [5], trained from known pathogenic non-coding variants of Mendelian disorders and a set of putatively benign variants. As pathogenic set, we use 406 hand-curated variants already used in the prior ReMM version [1], reciprocally lifted to GRCh38 using UCSC liftOver (RRID:SCR_018160) v377 [6] and validated for identical allelic sequences. The proxy-benign set includes around 14 million of human-lineage-derived sequence alterations [7], which we filtered to non-coding sequence using Jannovar v0.36 [8] and RefSeq (RRID:SCR_003496) [9]. Restricting variants to non-coding only removes a small proportion of variants (0.7% and 1% for GRCh37) and the high imbalance with the pathogenic variant set is similar on both genome builds (14.8M and 13.9M proxy-benign variants for GRCh37 and GRCh38, respectively). Therefore, we kept parameters for hyperSMURF model training as determined in Smedley et al. 2016 (Supplementary Table 1).

Imbalance-aware model training

The hyperSMURF algorithm applies a special sampling technique essential for the highly imbalanced data of human pathogenic variants [1,5]. The minority class (for ReMM the pathogenic variants) is oversampled based on the Synthetic Minority Over-sampling Technique (SMOTE) that creates synthetic examples using k-nearest neighbors rather than over-sampling the data with replacement [11]. The majority class (proxy-benign set) is divided into *n* non-overlapping partitions, which then are subsampled according to a ratio parameter. The minority class is oversampled by factor 2 and the majority class is undersampled by factor 3 which leads to the ration of pathogenic vs. benign variants of 2-3 in a more balanced dataset with around 2,000 datapoints. However, each balanced dataset alone provides insufficient coverage of the large data space of the majority class. That is why hyperSMURF applies an ensemble method: it divides the dataset into 100 partitions each containing all oversampled pathogenic and one partition of downsampled proxy-benign variants. On each partition, a random forest [12] is trained and the final pathogenicity score is the average over the 100 predictions. It ranges from 0 (not pathogenic) to 1 (pathogenic) and gives the probability values of a variant to belong to the pathogenic training data. Thus, the higher the score, the more likely that a variant at that position is pathogenic. We used parSMURF as implementation of hyperSMURF, a fast and highly scalable model training tool based on random forest algorithms [10].

Cytogenic band-aware cross-validation

Genomic data is confounded by local correlation of annotations, i.e., gnomically proximal variants tend to be more similar in their annotation results than random variants. Further, known pathogenic variants are not distributed evenly across the genome (e.g., due to selection bias, shared identification, available validation assays), but rather cluster around certain well-studied genes and share certain molecular functions or properties. When not accounted for, learners might infer superior hold-out performance because of genomic proximity of variants. To handle the local correlation structure in the genome, we apply ten-fold cytogenic band-aware cross-validation (CV) [1]. This is a stratified CV approach where each cytoband of the genome is associated to one out of ten folds. Folds are assigned to have a similar number of pathogenic variants, cytobands without pathogenic variants are randomly assigned to a fold. Proxy-benign variants are considered in the folds of their associated bands. Thereby gnomically proximal (i.e., same cytoband) pathogenic and proxy-benign variants are considered together, making it more challenging for the learner to discriminate between the two groups. Ten separate models are trained on nine folds and validated on the tenth fold. Unbiased predictions of variants contained in the training set can be performed with the model that only used the variants in the validation fold, while other variants are reported as the average of the ten model predictions.

Model features and imputation

Twenty-six selected features (see Supplementary Table 2) capture functional constraint and different sequence functions (sequence composition, epigenetics, conservation, population variance and regulatory regions) of the genetic variants. The feature set was kept close to the original feature set of ReMM, but some were not available from the original databases or were updated. Some features have a high proportion of missing values and the initial version of ReMM imputed all of them with zero. In genomics, a missing value often indicates an experimental signal that is too low to be measured, in line with this imputation. We have now identified some features (e.g., GC content or

conservation scores) where the genome-wide average of the annotation is more appropriate and impute them differently in this version (see Supplementary Table 2). For missing p-values, we use the value 1.

Availability of pre-scored files and scoring workflow
Pre-scored, block-gzip compressed and indexed whole genome files [13] were generated to allow a fast scoring of variants as well as an easy integration into other software. Every genomic position was scored with a general ReMM model trained on all data (v0.4.hg19 and v0.4.hg38, respectively). To guarantee unbiased score usage, e.g., for performance benchmarks with other tools, we replaced the score of variants in the training set with cross-validated scores (see above). The training and scoring pipeline is implemented in snakemake, a workflow management system for reproducible and scalable analysis [14,15].

ClinVar dataset
Version 2022-12-03 of NCBI ClinVar (RRID:SCR_006169) was downloaded on December 19th, 2022. Variants were filtered for single nucleotide changes with unambiguous clinical assertions of 'pathogenic', 'likely pathogenic', 'likely benign' and 'benign'. The set was annotated using Jannovar as described above and filtered for non-coding effects. Variants overlapping the training data set as well as mitochondrial SNVs were excluded (remaining n=946 likely pathogenic/pathogenic and n=192,057 likely benign/benign).

Comparison with other scores
For performance comparison on the GRCh38 training set (CV results as described above) of ReMM v0.4.hg38 with other scores, pre-scored GRCh38 whole genome files of CADD [7] version 1.6 were used to retrieve raw scores. ExPecto [16] and Sei [17] scores were computed using VCF files as described on their source code repositories ([18,19]). For ExPecto, the UCSC hg19 fasta reference file was replaced with hg38 to retrieve scores on the new genome build. Sei was run with the --hg38 option, respectively. The absolute mean and absolute maximum over all 218 outputs are used as final scores of Expecto. On the NCBI ClinVar set, we used GRCh37 whole-genome files of CADD v1.3, CADD v1.6. [7], ExPecto [18] and Sei [19] as described above, but with the UCSC hg19 reference genome. LINSIGHT scores [20] were downloaded from its source code repository [21] in bigWig format and extracted using the pyBigWig package [22]. fathmm-MKL [23] and RegBase [24] were downloaded in VCF format from the respective source code repositories [25], the scores were extracted using bcftools intersect [26]. The ncER v2 [27] bed file was downloaded from its dataset repository [28] using bedtools intersect to retrieve scores. ReMM scores for hg38 were included in the comparison by lifting the variant positions from the ClinVar set to the hg38 reference genome using UCSC liftOver (v377) [6] and extracting the corresponding ReMM v0.4.hg38 scores from the whole-genome file.

Results

Performance of ReMM on GRCh38
After 100 training cycles using different random seeds and ten-fold cytoband cross validation, we achieve a performance with an average area under the precision recall curve (AUPRC) of 0.613±0.005 (Supplementary Table 3). We randomly picked one model for the final scoring with an AUPRC of 0.610 (Figure 1a, receiver operating characteristic (ROC) performance available in Figure 1b).

Rather than using ReMM scores for ranking, some users choose to specify score thresholds for classifying into pathogenic and benign variants. Using a cutoff of 0.5 yields a good result in terms of retrieving known pathogenic non-coding variants (i.e., recall or True Positive rate, TP), but the number of benign variants might be extremely large. For ReMM v0.4.hg38, recall is 92% (375 out of 406) at a cutoff of 0.5 (Figure 1c), but precision is close to zero with a high false positive rate (FP) (86,507 out of 13,911,061; FP rate=0.006). The F1-score (harmonic mean of recall and precision) is highest at 0.963, resulting in a TP rate of 0.554 and a FP rate of 5.3e-6. Using the F2-score, we can give more weight to recall. Here, the optimal cutoff is 0.914, resulting in a TP rate of 0.702 and a FP rate of 2.3e-5. Analogous to NCBI ClinVar [29] pathogenic and likely pathogenic categories, we suggest to use a ReMM score above the F1 threshold as weak computational evidence for "pathogenic" and a score above the F2 threshold and below the F1 threshold for "likely pathogenic". For ReMM v0.4.hg19, these thresholds are 0.961 and 0.924 (Figure 1d), respectively.

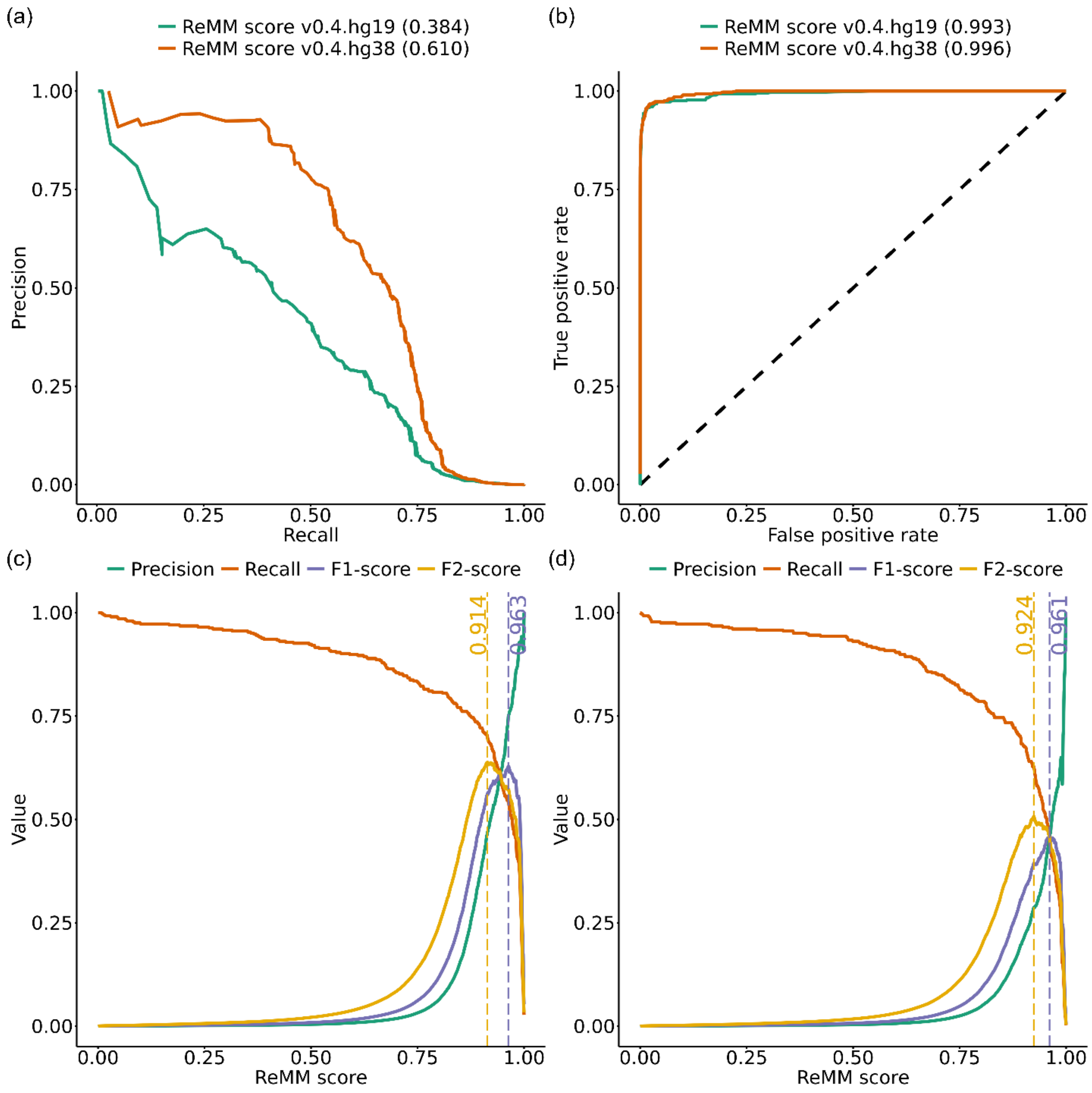

Figure 1: Precision, Recall, ROC, F1-, and F2-score curves – Performance metrics of ReMM v0.4.hg19 and v0.4.hg38 generated via ten-fold cytoband cross validation. Precision-Recall curves **(a)**, receiver operating characteristic (ROC) curves **(b)**, and precision, recall F1-score and F2-score (y-axis) over different ReMM score thresholds (x-axis) for v0.4.hg38 **(c)** and v0.4.hg19 **(d)**. Vertical lines denote the ReMM score with the maximum F1-score (yellow) and the maximum F2-score (purple). Area under the curve is shown in parentheses.

Correlation of scores and features

To compare both genome builds, we correlate ReMM scores from three genomic regions without assembly gap changes (DLK1, HBB, and PRDM9 loci) and >100,000 randomly sampled autosomal positions with successful reciprocal liftover (Supplementary Table 4). Here, ReMM scores are highly correlated between versions (Spearman and Pearson correlation between 0.7 and 0.8, Supplementary Table 4). We also used these regions and sites to explore the average feature correlation and find those to be similar, but except for one region (PRDM9) to be lower (Spearman correlation of 0.7 and Pearson correlation between 0.6-0.8, Supplementary Table 5). Further, we compare feature correlations between the genome builds directly on the training data (Figure 2). As expected from the high sequence similarity between reference sequence versions, we see the highest correlation for sequence features, like GC content.

Further, population variance features correlate well, with reduced correlation for the rare variant feature. This is likely due to spurious calls highly depending on the caller and the quality of the reference genome. We see the lowest correlation on the sparse Fantom5 regulatory element annotation data.

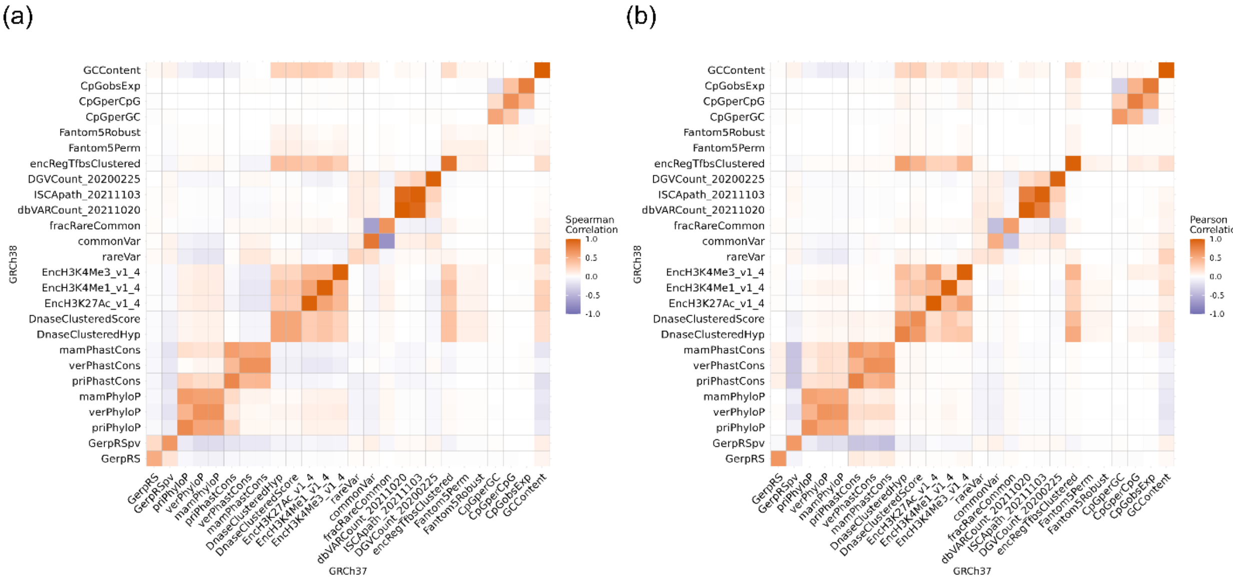

Figure 2: Correlation of feature values across genome builds – Feature correlation between features of the GRCh37 (x-axis) and the GRCh38 (y-axis) genome builds. The left heat map **(a)** shows Spearman correlation, and the right **(b)** shows Pearson correlation. Both plots show unexpectedly low correlations for some features along the diagonal. For example, the histone modification features (ENCODE) are lowly correlated as well as the enhancer features (FANTOM).

Imputing missing values

In previous ReMM versions, we used zero for missing values globally and trusted in the non-linearity of decision trees. Now, we use the average value of all defined positions for sequence and conservation features and one for p-values (see Supplementary Table 2). With the new approach, we see that the average AUPRC increases slightly (0.005 for v0.4.hg19, 0.009 for v0.4.hg38, Supplementary Table 6).

Feature importance

From the underlying Ranger random forest (RF) models [30], we retrieve feature importance using the Gini index. We averaged values over all 100 RFs in the model (Supplementary Table 7). In general, mean feature importance scores indicate contributions of all 26 features. No single feature stands out and our broad feature categories are all represented with at least one highly ranked feature. We interpret this as evidence that features were carefully picked and biases avoided. Epigenetic features increased in importance for the GRCh38 model (average rank 16 vs 19), may be due to better mapping and processing of the underlying data. Fantom5 features are probably too sparse to receive high importance but might be relevant for some variants. Between genome builds, feature importance values are similar, and no significant changes are detected (p-value 0.565, two-sided ranksum test). The replaced encRegTfbsClustered feature achieves a similar average Gini index (rank 6 on v0.4.hg19) as the previous numTFBSConserved feature (rank 4, data not shown).

Comparison to other scores

A number of different tools for scoring pathogenicity of non-coding variants exist [31]. However, a majority of tools are still based on the GRCh37 genome build which comes with the previously discussed drawbacks when scores are lifted to a new genome build[3,4,32]. To our knowledge, CADD, one of the most popular whole genome scores, seems to be the only tool directly trained on training data and features derived from GRCh38. Nine years after the GRCh38 release, no other non-coding score is adapted to the new genome build. Also, more recent sequence-based tools, like ExPecto [16] or DeepSEA-Sei [17] are trained on the previous genome release. However, coordinate liftover can be avoided for those tools because predictions are solely based on sequence and the sequence around variants from GRCh38 can be used directly. We compared performance of ReMM with CADD v1.6 GRCh38, Sei, and ExPecto on

the GRCh38 imbalanced training data. The area under the precision recall (PR) curve of ReMM substantially outperforms other methods (Figure 3a), while the area under the ROC curve is above 0.8 for all tools (Figure 3b). In the context of extremely unbalanced data, the area under the PR curve is more informative than the area under the ROC curve [33]. In these figures, the number of variants varies depending on how many were annotated with the respective tools. Specifically, ExPecto annotated only 7,299,993 out of the 14 million proxy-benign variants, probably due to missing transcripts close-by. Therefore, its performance might be overestimated. In Supplementary Figure 1, PR and ROC curves from the intersection of variants scored by all tools are shown (406 pathogenic and 7,299,993 proxy-benign variants), confirming that order and general result are stable despite the difference in the number of scored variants.

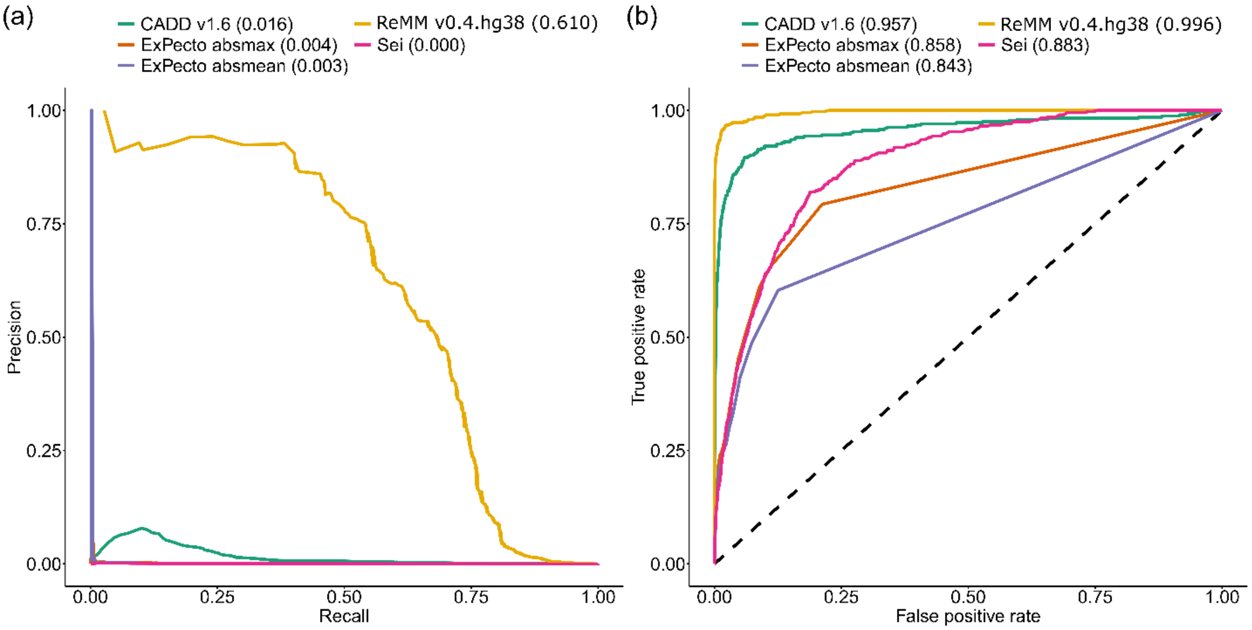

Figure 3: ROC and PR curve of ReMM, CADD, ExPecto, and Sei – Precision-Recall (PR) curves **(a)** and receiver operating characteristic (ROC) curves **(b)** of ReMM v0.4.hg38 (ten-fold cytoband cross validation scores) as well as CADD v1.6, ExPecto, and Sei on the GRCh38 training data. Area under the curve is shown in parentheses. ExPecto absmax is the maximum absolute value over all ExPecto outputs and ExPecto absmean the mean absolute value, respectively.

Due to the very limited availability of non-coding scores on GRCh38, we compared ReMM on GRCh37 with multiple other scores and on a set of non-coding variants from NCBI ClinVar that do not overlap its training set. We only used variants where all scores were able to provide a prediction (869 pathogenic and 190,548 benign) and plotted PR and ROC curves (Figure 4a and Figure 4b). CADD v1.6 achieved the best performance in terms of AURPC (0.160) and area under the ROC curve (AUROC, 0.811) followed by the (liftover) GRCh38 version of ReMM (AUPRC=0.035, AUROC=0.694). Interestingly, CADD v1.3, a previous version that does not yet include features for intronic splice variants, has a much lower performance on the non-coding ClinVar dataset (AUPRC=0.012, AUROC=0.633). We therefore speculate, that the performance boost in CADD v1.6 is due to the presence of many splice variants in the non-coding ClinVar dataset and the inclusion of specific splice-scores, like SpliceAI [34] or MMSsplice [35], in recent CADD versions. All compared scores, excluding CADD v1.6, are not optimized for splicing effects. Further, ReMM's pathogenic training set does neither contain splice variants nor did we add specific splicing features with this update.

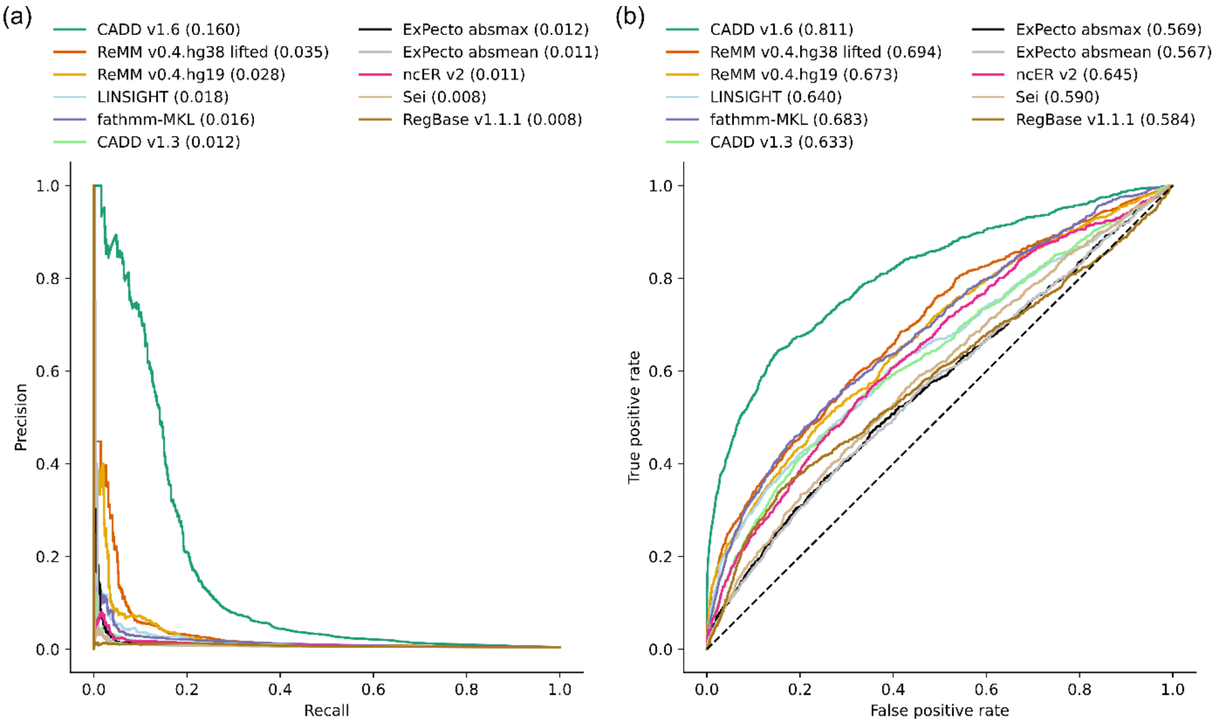

Figure 4: ROC and PR curve of on non-coding NCBI ClinVar – Precision-Recall (PR) curves **(a)** and receiver operating characteristic (ROC) curves **(b)** of different pathogenicity scores on NCBI pathogenic/likely pathogenic and benign/likely benign variants in the non-coding genome (absent from the training set of ReMM). All scores are trained and available on GRCh37. For ReMM v0.4.hg38 lifted, we lifted the GRCh37 ClinVar positions using UCSC liftOver and looked up the corresponding ReMM v0.4.hg38 score. Area under the curve is shown in parentheses and score names are sorted descending on the area under the PR curve. ExPecto absmax is the maximum absolute value over all ExPecto outputs and ExPecto absmean the mean absolute value, respectively.

Conclusion

The ReMM v0.4 score is a fully retrained non-coding score available for both the GRCh37 and GRCh38 genome builds. This fills the high need of supporting variant prioritization on the GRCh38 genome release, which is the *de facto* standard in research and routine diagnostics. Scores over the GRCh38 genome are highly correlated to the prior release with a performance increase for GRCh38 due to the better coverage of features. On imbalanced data (commonly observed in whole genome sequencing of individuals affected with Mendelian disease), ReMM scores outperform other non-coding effect scores. However, our analysis of new non-coding ClinVar variants also highlights limitations when scores are applied to variants (here splice variants) missing from the training data or for which no specific model features were included. In summary, we established a reproducible and scalable framework for integration of new features or new training data for further development of ReMM. The pre-scored whole genome files, UCSC genome browser annotation tracks [36] and a website provide fast access and easy usage of the ReMM score for researchers in all areas. With this release, tools like Genomiser [1] can now be run on the latest genome build, a highly demanded feature from the community.

AVAILABILITY OF SUPPORTING SOURCE CODE AND REQUIREMENTS

Project name: ReMM score

Project home page: https://remm.bihealth.org

Operating system(s): Platform independent (website), Linux (workflow)

Programming language: Python, Java, C++, Bash

Other requirements: browser (website); conda, snakemake, parSMURF (workflow)

License: MIT License

RRID:SCR_023095

DATA AVAILABILITY

We precomputed ReMM scores for all sequence-resolved positions in the genome (GRCh37 and GRCh38 builds) and provide them on Zenodo [37] or on the ReMM website [38], where we enable fast and easy scoring of variants. Variants can be uploaded via a VCF file [39], or scores directly displayed with a single site or genomic range variant lookup. Usage of UCSC genome browser tracks [36] of ReMM scores is described on the same website. In addition, we provide a REST-API that allows tools and scripts to retrieve ReMM scores directly. Scoring on the website is available for both genome builds and all major ReMM versions. ReMM is registered at bio.tools (biotools:remm_score) and has a Research Resource Identification Initiative ID (RRID:SCR_023095). The snakemake workflow to generate features, train scores and generate whole genome files is available on GitHub [40] or on WorkflowHub [14]. All supporting data are available in the *GigaScience* GigaDB database [41].

DECLARATIONS

List of abbreviations

AUPRC: area under the precision recall curve
AUROC: area under the receiver operating characteristic curves
API: Application Programming Interface
CADD: Combined Annotation-Dependent Depletion
CV: cross-validation
ENCODE: Encyclopedia of DNA elements
FANTOM: Functional annotation of the mammalian genome
GC: Guanine and Cytosine nucleotides in a sequence
GRCh: Genome Reference Consortium for Human
PR: precision recall curve
ReMM: Regulatory Mendelian Mutation
REST: Representation state transfer
RF: random forest
RRID: Research Resource Identification Initiative ID
ROC: receiver operating characteristic
SMOTE: Synthetic Minority Over-sampling Technique
UCSC: University of California, Santa Cruz
VCF: Variant Call Format

Ethics approval and consent to participate

Not applicable.

Consent for publication

Not applicable.

Competing interests

The authors declare that they have no competing interests.

Funding

Lusiné Nazaretyan is funded by the Helmholtz Einstein International Berlin Research School in Data Science (HEIBRiDS).

Authors' contributions

All authors designed the study. MS and LN prepared and analyzed the data. MS and LN wrote the software. All authors wrote the manuscript. All authors read and approved the submitted manuscript.

Acknowledgements

We thank current and previous members of the Kircher laboratory for helpful discussions and suggestions. Computation has been performed on the HPC for Research cluster of the Berlin Institute of Health at Charité – Universitätsmedizin Berlin.

REFERENCES

1.Smedley D, Schubach M, Jacobsen JOB, Köhler S, Zemojtel T, Spielmann M, et al. A Whole-Genome Analysis Framework for Effective Identification of Pathogenic Regulatory Variants in Mendelian Disease. Am J Hum Genet. 2016; doi:10.1016/j.ajhg.2016.07.005.
2.Guo Y, Dai Y, Yu H, Zhao S, Samuels DC, Shyr Y. Improvements and impacts of GRCh38 human reference on high throughput sequencing data analysis. Genomics. 2017; doi:10.1016/j.ygeno.2017.01.005.
3.Zheng-Bradley X, Streeter I, Fairley S, Richardson D, Clarke L, Flicek P, et al. Alignment of 1000 Genomes Project reads to reference assembly GRCh38. GigaScience. 2017; doi:10.1093/gigascience/gix038.
4.Wagner J, Olson ND, Harris L, McDaniel J, Cheng H, Fungtammasan A, et al. Curated variation benchmarks for challenging medically relevant autosomal genes. Nat Biotechnol. 2022; doi:10.1038/s41587-021-01158-1.
5.Schubach M, Re M, Robinson PN, Valentini G. Imbalance-Aware Machine Learning for Predicting Rare and Common Disease-Associated Non-Coding Variants. Sci Rep. 2017; doi:10.1038/s41598-017-03011-5.
6.Lee BT, Barber GP, Benet-Pagès A, Casper J, Clawson H, Diekhans M, et al. The UCSC Genome Browser database: 2022 update. Nucleic Acids Res. 2022; doi:10.1093/nar/gkab959.
7.Rentzsch P, Witten D, Cooper GM, Shendure J, Kircher M. CADD: predicting the deleteriousness of variants throughout the human genome. Nucleic Acids Res. 2019; doi:10.1093/nar/gky1016.
8.Jäger M, Wang K, Bauer S, Smedley D, Krawitz P, Robinson PN. Jannovar: A Java Library for Exome Annotation. Hum Mutat. 2014; doi:10.1002/humu.22531.
9.O'Leary NA, Wright MW, Brister JR, Ciufo S, Haddad D, McVeigh R, et al. Reference sequence (RefSeq) database at NCBI: current status, taxonomic expansion, and functional annotation. Nucleic Acids Res. 2016; doi:10.1093/nar/gkv1189.
10.Petrini A, Mesiti M, Schubach M, Frasca M, Danis D, Re M, et al. parSMURF, a high-performance computing tool for the genome-wide detection of pathogenic variants. GigaScience. 2020; doi:10.1093/gigascience/giaa052.
11.Chawla NV, Bowyer KW, Hall LO, Kegelmeyer WP. SMOTE: Synthetic Minority Over-sampling Technique. J Artif Intell Res. 2002; doi:10.1613/jair.953.
12.Breiman L. Random Forests. Mach Learn. 2001; doi:10.1023/A:1010933404324.
13.Li H. Tabix: fast retrieval of sequence features from generic TAB-delimited files. Bioinformatics. 2011; doi:10.1093/bioinformatics/btq671.
14.Schubach M. ReMM score. WorkflowHub; 2023; doi:10.48546/workflowhub.workflow.414.1.
15.Mölder F, Jablonski KP, Letcher B, Hall MB, Tomkins-Tinch CH, Sochat V, et al. Sustainable data analysis with Snakemake. F1000Research. 2021; doi:10.12688/f1000research.29032.2.
16.Zhou J, Theesfeld CL, Yao K, Chen KM, Wong AK, Troyanskaya OG. Deep learning sequence-based ab initio prediction of variant effects on expression and disease risk. Nat Genet. Nature Publishing Group; 2018; doi:10.1038/s41588-018-0160-6.
17.Chen KM, Wong AK, Troyanskaya OG, Zhou J. A sequence-based global map of regulatory activity for deciphering human genetics. Nat Genet. Nature Publishing Group; 2022; doi:10.1038/s41588-022-01102-2.
18. ExPecto (2018). ExPecto https://github.com/FunctionLab/ExPecto
19. Sei framework (2022). Sei framework https://github.com/FunctionLab/sei-framework
20.Gronau I, Arbiza L, Mohammed J, Siepel A. Inference of natural selection from interspersed genomic elements based on polymorphism and divergence. Mol Biol Evol. 2013; doi:10.1093/molbev/mst019.
21.Siepel A. CshlSiepelLab/LINSIGHT. https://github.com/CshlSiepelLab/LINSIGHT Accessed 15 Mar 2023.
22.Ryan D, Grüning B, Ramirez F. Pybigwig 0.2.4 (2016). Pybigwig 0.2.4 Zenodo; http://zenodo.org/record/45238
23.Shihab HA, Rogers MF, Gough J, Mort M, Cooper DN, Day INM, et al. An integrative approach to predicting the functional effects of non-coding and coding sequence variation. Bioinforma Oxf Engl. 2015; doi:10.1093/bioinformatics/btv009.
24.Zhang S, He Y, Liu H, Zhai H, Huang D, Yi X, et al. regBase: whole genome base-wise aggregation and functional prediction for human non-coding regulatory variants. Nucleic Acids Res. 2019; doi:10.1093/nar/gkz774.
25. regBase (2019). regBase https://github.com/mulinlab/regBase
26.Li H. A statistical framework for SNP calling, mutation discovery, association mapping and population genetical parameter estimation from sequencing data. Bioinformatics. 2011; doi:10.1093/bioinformatics/btr509.

27. Wells A, Heckerman D, Torkamani A, Yin L, Sebat J, Ren B, et al. Ranking of non-coding pathogenic variants and putative essential regions of the human genome. Nat Commun. Nature Publishing Group; 2019; doi:10.1038/s41467-019-13212-3.
28. TelentiLab/ncER_datasets. https://github.com/TelentiLab/ncER_datasets Accessed 2 Mar 2023.
29. Landrum MJ, Lee JM, Benson M, Brown GR, Chao C, Chitipiralla S, et al. ClinVar: improving access to variant interpretations and supporting evidence. Nucleic Acids Res. 2018; doi:10.1093/nar/gkx1153.
30. Wright MN, Ziegler A. ranger: A Fast Implementation of Random Forests for High Dimensional Data in C++ and R. J Stat Softw. 2017; doi:10.18637/jss.v077.i01.
31. Wang Z, Zhao G, Li B, Fang Z, Chen Q, Wang X, et al. Performance comparison of computational methods for the prediction of the function and pathogenicity of non-coding variants. Genomics Proteomics Bioinformatics. 2022; doi:10.1016/j.gpb.2022.02.002.
32. Nurk S, Koren S, Rhie A, Rautiainen M, Bzikadze AV, Mikheenko A, et al. The complete sequence of a human genome. Science. American Association for the Advancement of Science; 2022; doi:10.1126/science.abj6987.
33. Saito T, Rehmsmeier M. The Precision-Recall Plot Is More Informative than the ROC Plot When Evaluating Binary Classifiers on Imbalanced Datasets. PLOS ONE. 2015; doi:10.1371/journal.pone.0118432.
34. Jaganathan K, Kyriazopoulou Panagiotopoulou S, McRae JF, Darbandi SF, Knowles D, Li YI, et al. Predicting Splicing from Primary Sequence with Deep Learning. Cell. 2019; doi:10.1016/j.cell.2018.12.015.
35. Cheng J, Nguyen TYD, Cygan KJ, Çelik MH, Fairbrother WG, Avsec Z, et al. MMSplice: modular modeling improves the predictions of genetic variant effects on splicing. Genome Biol. 2019; doi:10.1186/s13059-019-1653-z.
36. Kent WJ, Sugnet CW, Furey TS, Roskin KM, Pringle TH, Zahler AM, et al. The Human Genome Browser at UCSC. Genome Res. 2002; doi:10.1101/gr.229102.
37. Schubach M, Nazaretyan L, Kircher M. ReMM score. Zenodo. 2022. http://doi.org/10.5281/zenodo.6576087
38. Schubach M, Nazaretyan L, Kircher M. ReMM score: Regulatory Mendelian Mutation score. https://remm.kircherlab.bihealth.org/ Accessed 14 Mar 2023.
39. Danecek P, Auton A, Abecasis G, Albers CA, Banks E, DePristo MA, et al. The variant call format and VCFtools. Bioinformatics. 2011; doi:10.1093/bioinformatics/btr330.
40. Schubach M, Nazaretyan L. ReMM (2022). ReMM https://github.com/kircherlab/ReMM
41. Schubach M, Nazaretyan L, Kircher M. Supporting data for "The Regulatory Mendelian Mutation score for GRCh38." GigaScience Database. 2023. http://doi.org/10.5524/102376

Figure 1 Click here to access/download;Figure;Figure1.png

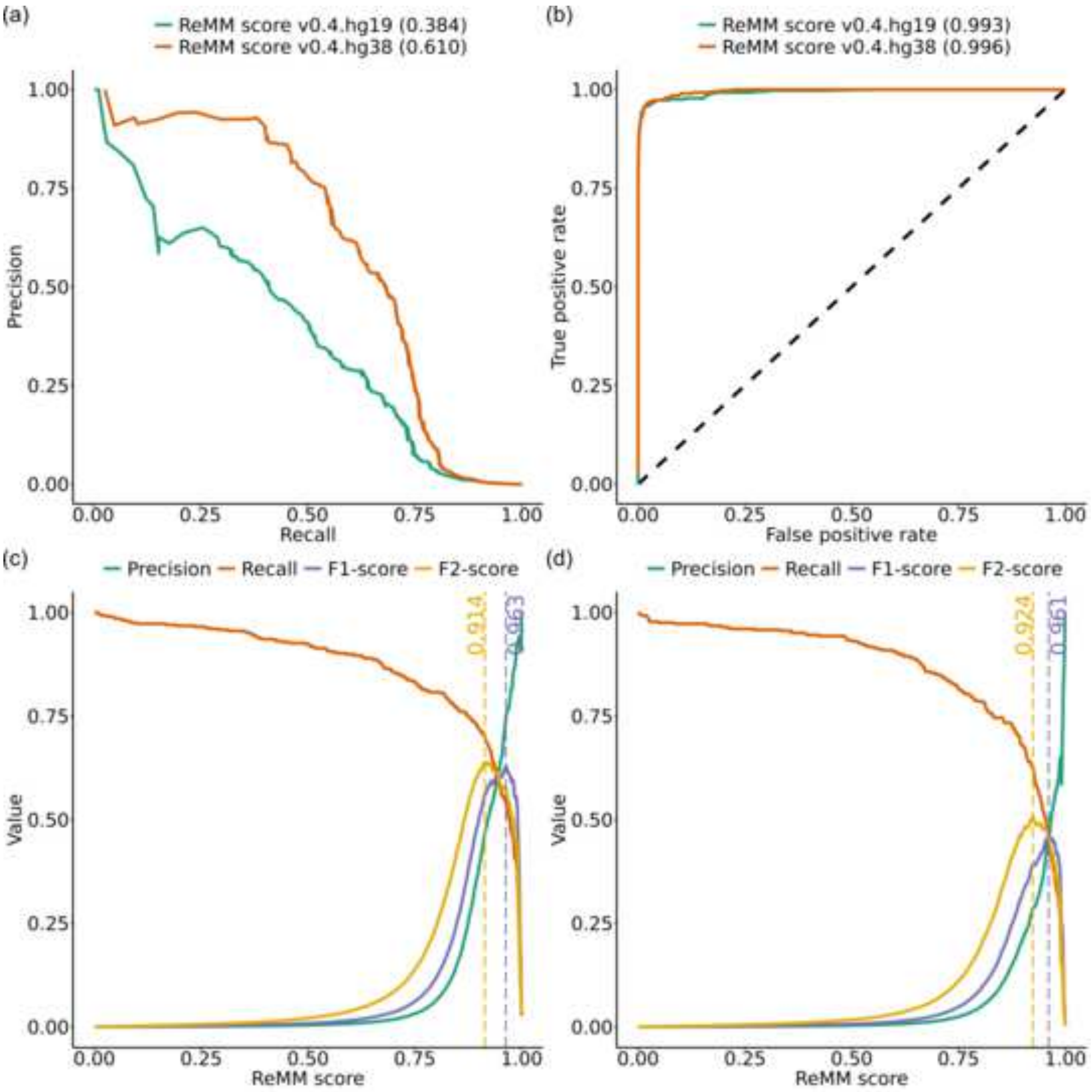

Figure 2

Click here to access/download;Figure;Figure2.png

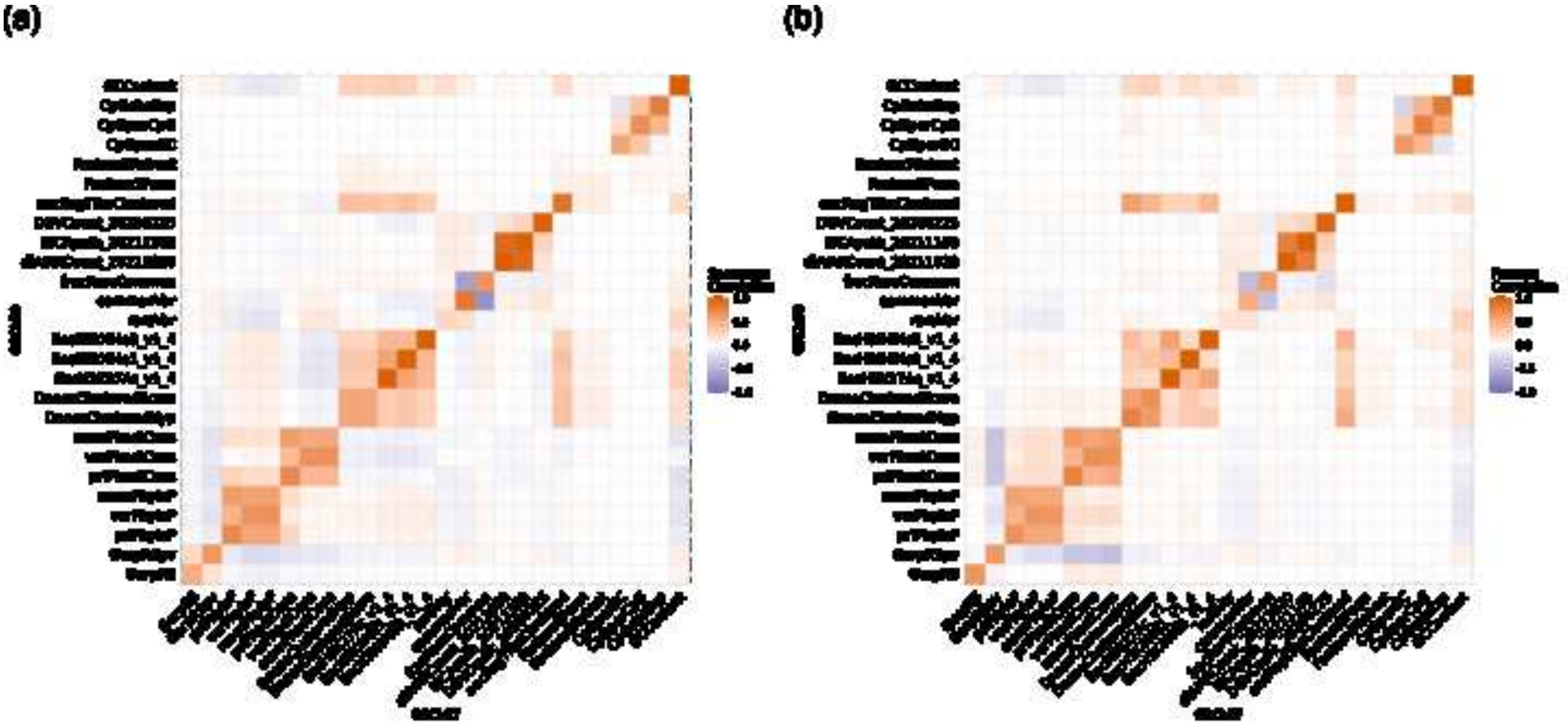

Figure 3

Click here to access/download;Figure;Figure3.png

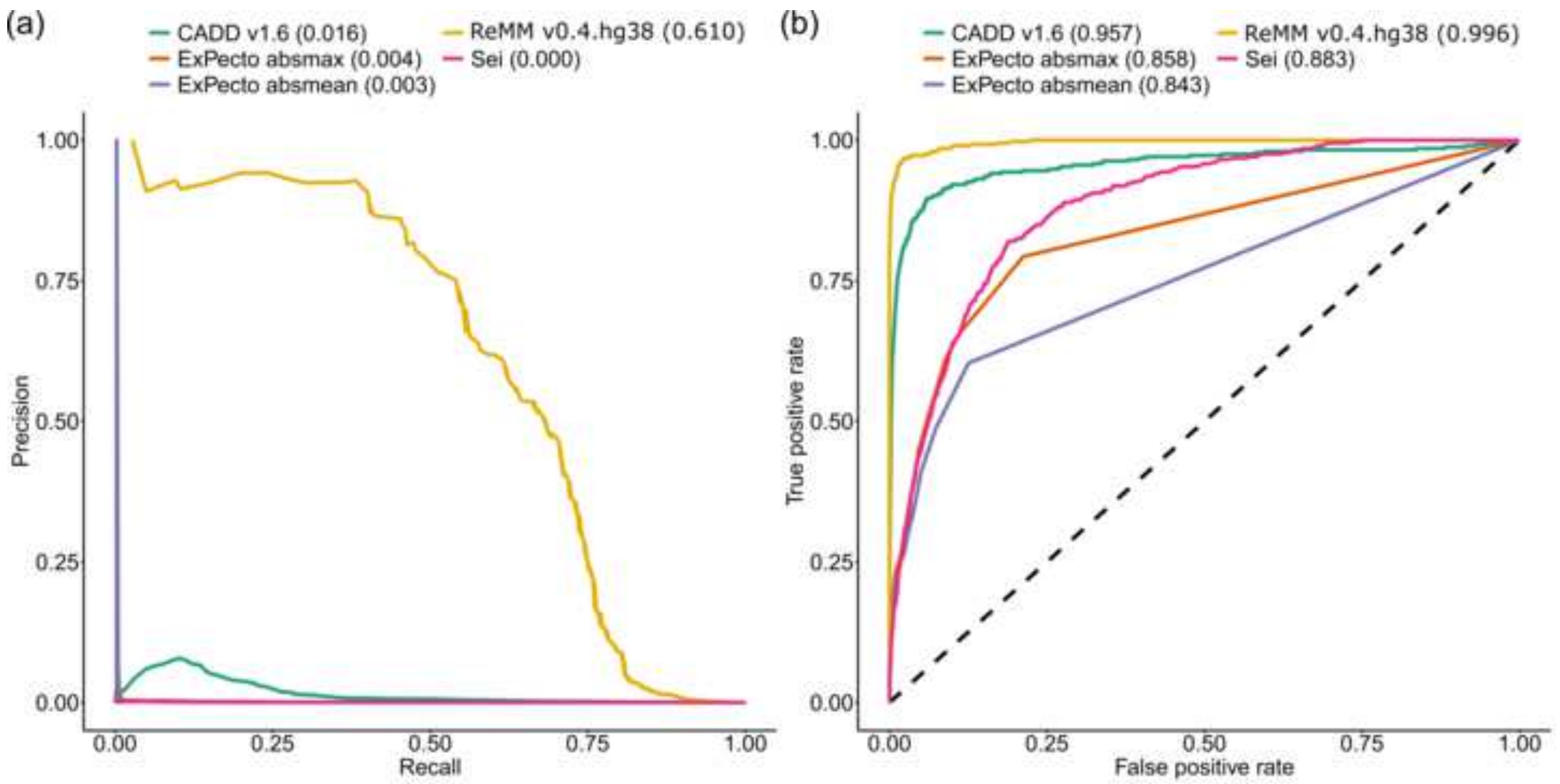

Figure 4

Click here to access/download;Figure;Figure4.png

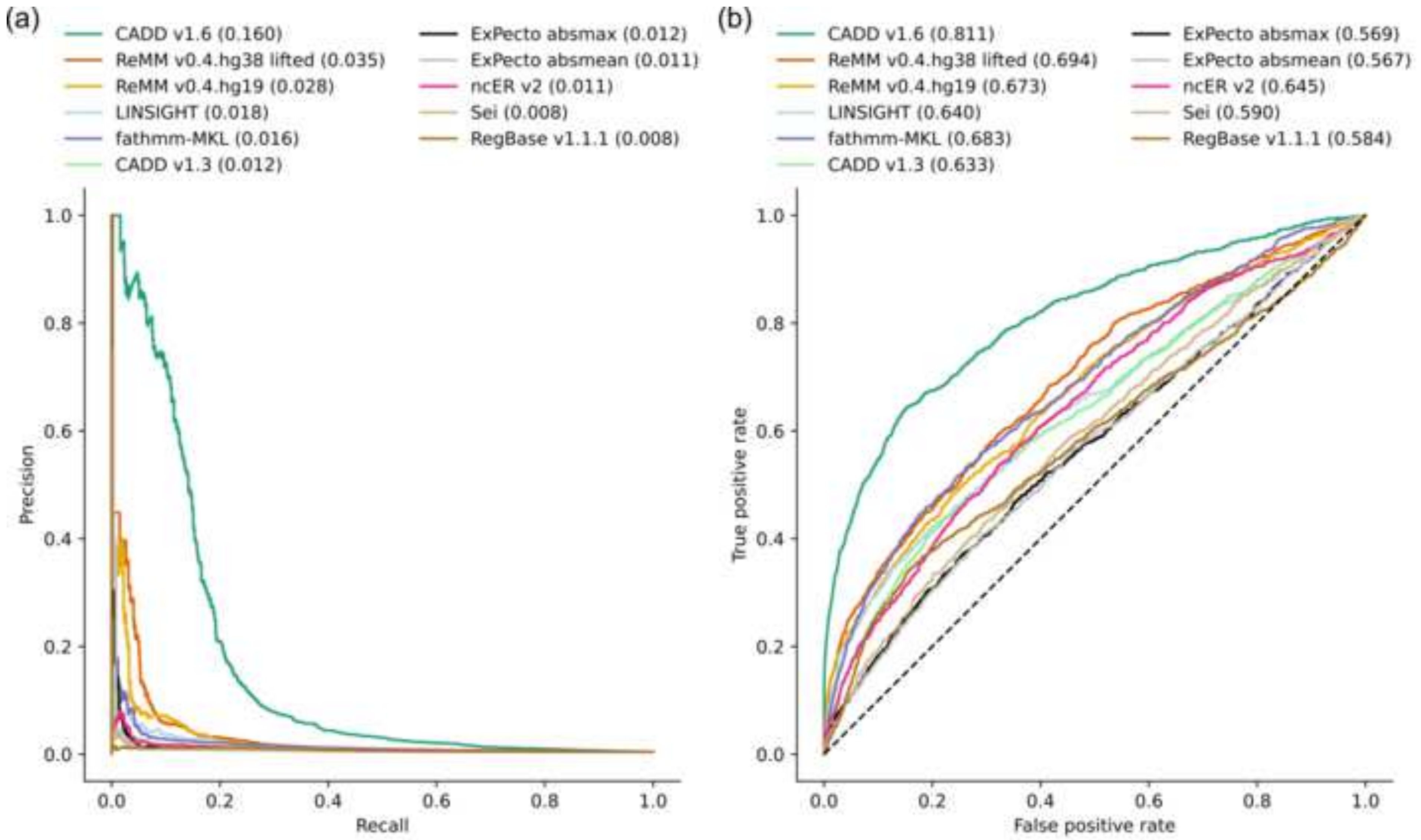

Supplementary Material

Click here to access/download
Supplementary Material
ReMM_Supplement.docx
