# Supplementary material for: The Regulatory Mendelian Mutation score for GRCh38
Source: Gigascience. 2023 Apr 21;12:giad024. doi: 10.1093/gigascience/giad024 (PMC10120424; doi:10.1093/gigascience/giad024)
Supplement: giad024_Supplemental_File [file giad024_supplemental_file.docx]

# Supplementary Material

The Regulatory Mendelian Mutation score for GRCh38

Max Schubach^1^_,_ Lusiné Nazaretyan^1^, Martin Kircher^1,2^

^1^ Berlin Institute of Health at Charité – Universitätsmedizin Berlin, Charitéplatz 1, Berlin, Germany
^2^ Institute of Human Genetics, University Medical Center Schleswig-Holstein, University of Lübeck, Ratzeburger Allee 160, Lübeck, Germany

## Supplementary Tables

Supplementary Table 1: Hyperparameters – Hyperparameters of ReMM training (used across all currently available versions).

| Parameter | Value | Description |
| --- | --- | --- |
| $n$ | 100 | Number of partitions |
| $o$ | 2 | SMOTE oversampling factor |
| $k$ | 5 | SMOTE k-nearest neighbor |
| $k$ | 3 | Undersampling factor |
| $t$ | 10 | Forest size for each Random Forest |
| $d$ | 5 | Random tree features for each Random Forest |

Supplementary Table 2: Features – Features used for training of the ReMM v0.4 score and their default for missing values. The description column contains the original source from where features were downloaded.

| Feature | Missing value (hg19/hg38) | Description |
| --- | --- | --- |
| CpGperGC | 65.085/ 65.8818 | Percentage of CpG island that is C or G.  https://hgdownload.soe.ucsc.edu/goldenPath/hg38/database/cpgIslandExt.txt.gz (hg19)  https://hgdownload.soe.ucsc.edu/goldenPath/hg38/database/cpgIslandExt.txt.gz (hg38) |
| CpGperCpG | 18.2498/  18.7278 | Percentage of CpG island that is CpG.  https://hgdownload.soe.ucsc.edu/goldenPath/hg38/database/cpgIslandExt.txt.gz (hg19)  https://hgdownload.soe.ucsc.edu/goldenPath/hg38/database/cpgIslandExt.txt.gz (hg38) |
| CpGobsExp | 0.874298/  0.874696 | Ratio of observed to expected CpGs in CpG island.  https://hgdownload.soe.ucsc.edu/goldenPath/hg38/database/cpgIslandExt.txt.gz (hg19)  https://hgdownload.soe.ucsc.edu/goldenPath/hg38/database/cpgIslandExt.txt.gz (hg38) |
| GCContent | 0.409034/  0.408699 | GC-content in a window of ±75 bp of the reference genome |
| DnaseClusteredHyp | 0.0/0.0 | DnaseClustered (V3) hypersensitivity score  https://hgdownload.soe.ucsc.edu/goldenPath/hg19/database/wgEncodeRegDnaseClusteredV3.txt.gz (hg19)  https://hgdownload.soe.ucsc.edu/goldenPath/hg38/database/wgEncodeRegDnaseClustered.txt.gz (hg38) |
| DnaseClusteredScore | 0.0/0.0 | Number of DnaseClustered (V3) hypersensitive cells https://hgdownload.soe.ucsc.edu/goldenPath/hg19/database/wgEncodeRegDnaseClusteredV3.txt.gz (hg19)  https://hgdownload.soe.ucsc.edu/goldenPath/hg38/database/wgEncodeRegDnaseClustered.txt.gz (hg38) |
| EncH3K27Ac | 0.0/0.0 | Maximum ENCODE H3K27 acetylation level  https://hgdownload.soe.ucsc.edu/gbdb/hg19/bbi/wgEncodeBroadHistone{Gm12878,H1hesc,Hsmm,Huvec,K562,Nhek,Nhlf}H3k27acStdSig.bigWig (hg19)  https://hgdownload.soe.ucsc.edu/gbdb/hg38/bbi/wgEncodeReg/wgEncodeRegMarkH3k27ac/wgEncodeBroadHistone{Gm12878,H1hesc,Hsmm,Huvec,K562,Nhek,Nhlf}H3k27acStdSig.bigWig (hg38) |
| EncH3K4Me1 | 0.0/0.0 | Maximum ENCODE H3K4 methylation level  https://hgdownload.soe.ucsc.edu/gbdb/hg19/bbi/wgEncodeBroadHistone{Gm12878,H1hesc,Hsmm,Huvec,K562,Nhek,Nhlf}H3k4me1StdSig.bigWig (hg19)  https://hgdownload.soe.ucsc.edu/gbdb/hg38/bbi/wgEncodeReg/wgEncodeRegMarkH3k27ac/wgEncodeBroadHistone{Gm12878,H1hesc,Hsmm,Huvec,K562,Nhek,Nhlf}H3k4me1StdSig.bigWig (hg38) |
| EncH3K4Me3 | 0.0/0.0 | Maximum ENCODE H3K4 methylation level  https://hgdownload.soe.ucsc.edu/gbdb/hg19/bbi/wgEncodeBroadHistone{Gm12878,H1hesc,Hsmm,Huvec,K562,Nhek,Nhlf}H3k4me3StdSig.bigWig (hg19)  https://hgdownload.soe.ucsc.edu/gbdb/hg38/bbi/wgEncodeReg/wgEncodeRegMarkH3k27ac/wgEncodeBroadHistone{Gm12878,H1hesc,Hsmm,Huvec,K562,Nhek,Nhlf}H3k4me3StdSig.bigWig (hg38) |
| Fantom5Perm | 0.0/0.0 | FANTOM 5 permissive enhancers  http://enhancer.binf.ku.dk/presets/permissive_enhancers.bed (hg19)  https://doi.org/10.5281/zenodo.545682 (hg38) |
| Fantom5Robust | 0.0/0.0 | FANTOM 5 robust enhancers  http://enhancer.binf.ku.dk/presets/robust_enhancers.bed (hg19)  https://doi.org/10.5281/zenodo.545682 (hg38) |
| encRegTfbsClustered | 0/0 | Number of ENCODE Regulation 'TF Clusters'  https://hgdownload.soe.ucsc.edu/goldenPath/hg19/database/encRegTfbsClustered.txt.gz (hg19)  https://hgdownload.soe.ucsc.edu/goldenPath/hg38/database/encRegTfbsClustered.txt.gz (hg38) |
| priPhyloP | 0.0451375/  0.0977878 | Primate PhyloP score  https://hgdownload.soe.ucsc.edu/goldenPath/hg19/phyloP46way/primates/ (hg19)  https://hgdownload.soe.ucsc.edu/goldenPath/hg38/phyloP17way/hg38.phyloP17way.wigFix.gz (hg38) |
| priPhastCons | 0.0977878/  0.151415 | Primate PhastCons conservation score  https://hgdownload.soe.ucsc.edu/goldenPath/hg19/phastCons46way/primates/ (hg19)  https://hgdownload.soe.ucsc.edu/goldenPath/hg38/phastCons17way/hg38.phastCons17way.wigFix.gz (hg38) |
| verPhyloP | 0.0892683/ 0.0954381 | Vertebrate PhyloP score  https://hgdownload.soe.ucsc.edu/goldenPath/hg19/phyloP46way/vertebrate/ (hg19)  https://hgdownload.soe.ucsc.edu/goldenPath/hg38/phyloP100way/hg38.100way.phyloP100way/ (hg38) |
| verPhastCons | 0.102729/ 0.0980657 | Vertebrate PhastCons conservation score  https://hgdownload.soe.ucsc.edu/goldenPath/hg19/phastCons46way/vertebrate/ (hg19)  https://hgdownload.soe.ucsc.edu/goldenPath/hg38/phastCons100way/hg38.100way.phastCons/ (hg38) |
| mamPhyloP | 0.0357644/ 0.100913 | Mammalian PhyloP score.  https://hgdownload.soe.ucsc.edu/goldenPath/hg19/phyloP46way/placentalMammals/ (hg19)  https://hgdownload.soe.ucsc.edu/goldenPath/hg38/phyloP30way/hg38.30way.phyloP/ (hg38) |
| mamPhastCons | 0.0878941/ 0.128491 | Mammalian PhastCons conservation score  https://hgdownload.soe.ucsc.edu/goldenPath/hg19/phastCons46way/placentalMammals/ (hg19)  https://hgdownload.soe.ucsc.edu/goldenPath/hg38/phastCons30way/hg38.30way.phastCons/ (hg38) |
| GerpRS | 1064.06/ 1441.64 | GERP++ element score  http://mendel.stanford.edu/SidowLab/downloads/gerp/hg19.GERP_elements.tar.gz (hg19)  From CADD v1.3 (hg38) |
| GerpRSpv | 1.0/1.0 | GERP++ element p-Value  http://mendel.stanford.edu/SidowLab/downloads/gerp/hg19.GERP_elements.tar.gz (hg19)  From CADD v1.3 (hg38) |
| rareVar | 0/0 | Number of rare 1KG variants (≤ 5% AF) in a window of ±500 bp  http://ftp.1000genomes.ebi.ac.uk/vol1/ftp/data_collections/1000G_2504_high_coverage/working/20200515_EBI_Freebayescalls (hg38) |
| commonVar | 0/0 | Number of common 1KG variants (> 5% AF) in a window of ±500 bp  http://ftp.1000genomes.ebi.ac.uk/vol1/ftp/data_collections/1000G_2504_high_coverage/working/20200515_EBI_Freebayescalls (hg38) |
| fracRareCommon | 0.0/0.0 | Ratio rare to common variants  http://ftp.1000genomes.ebi.ac.uk/vol1/ftp/data_collections/1000G_2504_high_coverage/working/20200515_EBI_Freebayescalls (hg38) |
| ISCApath | 0/0 | Overlapping ISCA CNVs (date 11/03/2021)  https://ftp.ncbi.nlm.nih.gov/pub/dbVar/data/Homo_sapiens/by_study/tsv/nstd46.GRCh37.variant_call.tsv.gz (hg19)  https://ftp.ncbi.nlm.nih.gov/pub/dbVar/data/Homo_sapiens/by_study/tsv/nstd75.GRCh37.variant_call.tsv.gz (hg19)  https://ftp.ncbi.nlm.nih.gov/pub/dbVar/data/Homo_sapiens/by_study/tsv/nstd102.GRCh37.variant_call.tsv.gz (hg19)  https://ftp.ncbi.nlm.nih.gov/pub/dbVar/data/Homo_sapiens/by_study/tsv/nstd46.GRCh38.variant_call.tsv.gz (hg38)  https://ftp.ncbi.nlm.nih.gov/pub/dbVar/data/Homo_sapiens/by_study/tsv/nstd75.GRCh38.variant_call.tsv.gz (hg38)  https://ftp.ncbi.nlm.nih.gov/pub/dbVar/data/Homo_sapiens/by_study/tsv/nstd102.GRCh38.variant_call.tsv.gz (hg38) |
| dbVARCount | 0/0 | Overlapping dbVAR CNVs (date 10/20/2021)  https://ftp.ncbi.nlm.nih.gov/pub/dbVar/archive/Homo_sapiens/by_assembly/GRCh37/gvf/GRCh37.2021_10_20.variant_call.clinical.pathogenic_or_likely_pathogenic.gvf.gz (hg19)  https://ftp.ncbi.nlm.nih.gov/pub/dbVar/archive/Homo_sapiens/by_assembly/GRCh38/gvf/GRCh38.2021_10_20.variant_call.clinical.pathogenic_or_likely_pathogenic.gvf.gz (hg38) |
| DGVCount | 0/0 | Overlapping DGV CNVs (date 02/25/2020)  http://dgv.tcag.ca/dgv/docs/GRCh37_hg19_variants_2020-02-25.txt (hg19)  http://dgv.tcag.ca/dgv/docs/GRCh38_hg38_variants_2020-02-25.txt (hg38) |

Supplementary Table 3: ReMM score v0.4 performance – Area under the precision recall curve (AUPRC) and area under the receiver-operating characteristic curve (AUROC) for ReMM score v0.4 on both genome builds, as well as average values (avg) with standard deviation in parentheses across 100 training runs. AUPRC and AUROC are computed via 10-fold cytoband cross-validation.

|  | GRCh38 | | GRCh37 | |
| --- | --- | --- | --- | --- |
|  | **Avg of 100 runs** | **ReMM score v0.4** | **Avg of 100 runs** | **ReMM score v0.4** |
| AUPRC | 0.613 (±0.005) | 0.610 | 0.384 (±0.014) | 0.394 |
| AUROC | 0.996 (±0.000) | 0.996 | 0.993 (±0.000) | 0.993 |

Supplementary Table 4: ReMM score correlation across genome builds – Pearson and Spearman correlation of ReMM scores between genome builds of three genic regions (DLK1, HBB, PRDM9) and 120,000 random positions (120K). For 120K, only variants with a successful coordinate liftOver from GRCh38 to GRCh37 and located on major human chromosomes are used (n=110,751).

| Name | Length (bps) | GRCh37 coordinates | GRCh38 coordinates | Pearson correlation | Spearman correlation |
| --- | --- | --- | --- | --- | --- |
| DLK1 | 123,945 | chr14:101,118,632-101,242,576 | chr14: 100,652,295-100,776,239 | 0.717 | 0.740 |
| HBB | 160,600 | chr11:5,167,199-5,327,798 | chr11:5,145,969-5,306,568 | 0.829 | 0.825 |
| PRDM9 | 106,589 | chr5:23,464,354-23,570,942 | chr5:234,64,245-23,570,833 | 0.685 | 0.692 |
| 120K | 110,751 | - | - | 0.774 | 0.779 |

Supplementary Table 5: Feature value correlations across genome builds for regions and variants – Average Pearson and Spearman correlation of the 26 features used for each genome build in three genic regions (DLK1, HBB, PRDM9) and 120,000 random positions (120K). For 120K, only variants with a successful coordinate liftOver from GRCh38 to GRCh37 and located on major human chromosomes are used (n=110,751).

| Name | Length (bps) | GRCh37 coordinates | GRCh38 coordinates | Pearson correlation | Spearman correlation |
| --- | --- | --- | --- | --- | --- |
| DLK1 | 123,945 | chr14:101,118,632-101,242,576 | chr14:100,652,295-100,776,239 | 0.680 | 0.686 |
| HBB | 160,600 | chr11:5,167,199-5,327,798 | chr11:5,145,969-5,306,568 | 0.713 | 0.708 |
| PRDM9 | 106,589 | chr5:23,464,354-23,570,942 | chr5:234,64,245-23,570,833 | 0.763 | 0.738 |
| 120K | 110,751 | - | - | 0.572 | 0.650 |

Supplementary Table 6: ReMM performance dependent on missing values – Average area under the precision recall curve (AUPRC) and area under the receiver-operating characteristic curve (AUROC) values with standard deviation in parentheses from 100 model training runs using zero as missing values or the default values listed in Supplementary Table 2. AUPRC and AUROC are computed via ten-fold cytoband cross-validation.

|  | **GRCh38** | | **GRCh37** | |
| --- | --- | --- | --- | --- |
|  | **Zero** | **Default value** | **Zero** | **Default value** |
| AUPRC | 0.594 (±0.007) | 0.613 (±0.005) | 0.379 (±0.015) | 0.384 (±0.014) |
| AUROC | 0.996 (±0.000) | 0.996 (±0.000) | 0.993 (±0.000) | 0.993 (±0.000) |

Supplementary Table 7: Feature importance – Average feature importance (Gini index) over 100 Random Forest partitions of the hyperSMURF models of ReMM v0.4.hg19 and v0.4.hg38. Gini index values were derived with the Ranger package after training on all training data. The standard deviation (std), the minimum (min) and the maximum (max) value across the 100 partitions is shown. The rank indicates the importance rank by average Gini index.

|  |  |  | **ReMM v0.4.hg38** | | | |  | **ReMM v0.4.hg19** | | | |
| --- | --- | --- | --- | --- | --- | --- | --- | --- | --- | --- | --- |
| **Category** | **Feature** | **rank** | **mean** | **std** | **min** | **max** | **rank** | **mean** | **std** | **min** | **max** |
| Conservation | GerpRS | 4 | 0.0258 | 0.0074 | 0.0119 | 0.0444 | 5 | 0.0287 | 0.0082 | 0.0134 | 0.0538 |
| Conservation | mamPhastCons | 7 | 0.0149 | 0.0051 | 0.0013 | 0.0254 | 25 | 0.0000 | 0.0000 | -0.0001 | 0.0002 |
| Conservation | mamPhyloP | 10 | 0.0118 | 0.0048 | 0.0031 | 0.0287 | 10 | 0.0096 | 0.0037 | 0.0022 | 0.0203 |
| Conservation | GerpRSpv | 16 | 0.0036 | 0.0020 | 0.0008 | 0.0134 | 17 | 0.0020 | 0.0014 | -0.0004 | 0.0114 |
| Conservation | verPhyloP | 18 | 0.0033 | 0.0018 | 0.0002 | 0.0103 | 15 | 0.0035 | 0.0018 | 0.0007 | 0.0133 |
| Conservation | priPhyloP | 20 | 0.0020 | 0.0023 | -0.0002 | 0.0141 | 13 | 0.0051 | 0.0029 | 0.0003 | 0.0136 |
| Conservation | verPhastCons | 21 | 0.0018 | 0.0008 | 0.0001 | 0.0048 | 16 | 0.0021 | 0.0010 | 0.0006 | 0.0055 |
| Conservation | priPhastCons | 25 | 0.0008 | 0.0018 | -0.0001 | 0.0177 | 14 | 0.0049 | 0.0031 | 0.0005 | 0.0186 |
| Epigenetic | EncH3K4Me1 | 12 | 0.0106 | 0.0032 | 0.0058 | 0.0186 | 8 | 0.0113 | 0.0031 | 0.0059 | 0.0217 |
| Epigenetic | EncH3K4Me3 | 13 | 0.0105 | 0.0103 | 0.0005 | 0.0551 | 22 | 0.0009 | 0.0012 | -0.0002 | 0.0078 |
| Epigenetic | DnaseClusteredHyp | 14 | 0.0091 | 0.0049 | 0.0004 | 0.0281 | 21 | 0.0012 | 0.0009 | -0.0001 | 0.0062 |
| Epigenetic | EncH3K27Ac | 19 | 0.0030 | 0.0028 | -0.0001 | 0.0121 | 26 | 0.0000 | 0.0000 | -0.0001 | 0.0001 |
| Epigenetic | DnaseClusteredScore | 24 | 0.0014 | 0.0013 | 0.0000 | 0.0087 | 19 | 0.0015 | 0.0008 | -0.0005 | 0.0042 |
| Population | dbVARCount | 1 | 0.1065 | 0.0203 | 0.0672 | 0.1652 | 1 | 0.1062 | 0.0159 | 0.0679 | 0.1385 |
| Population | commonVar | 2 | 0.0657 | 0.0177 | 0.0271 | 0.1044 | 3 | 0.0362 | 0.0140 | 0.0070 | 0.0746 |
| Population | fracRareCommon | 5 | 0.0167 | 0.0063 | 0.0047 | 0.0383 | 6 | 0.0252 | 0.0076 | 0.0116 | 0.0499 |
| Population | rareVar | 9 | 0.0124 | 0.0059 | 0.0023 | 0.0318 | 2 | 0.0366 | 0.0140 | 0.0082 | 0.0875 |
| Population | DGVCount | 15 | 0.0083 | 0.0044 | 0.0008 | 0.0201 | 7 | 0.0115 | 0.0052 | 0.0029 | 0.0293 |
| Population | ISCApath | 17 | 0.0035 | 0.0020 | 0.0002 | 0.0086 | 24 | 0.0007 | 0.0007 | -0.0002 | 0.0039 |
| Regulatory | encRegTfbsClustered | 6 | 0.0164 | 0.0074 | 0.0046 | 0.0390 | 4 | 0.0331 | 0.0120 | 0.0090 | 0.0664 |
| Regulatory | Fantom5Robust | 22 | 0.0015 | 0.0015 | 0.0003 | 0.0101 | 18 | 0.0017 | 0.0009 | 0.0004 | 0.0051 |
| Regulatory | Fantom5Perm | 23 | 0.0014 | 0.0008 | 0.0002 | 0.0053 | 20 | 0.0012 | 0.0014 | -0.0001 | 0.0116 |
| Sequence | CpGperGC | 3 | 0.0339 | 0.0106 | 0.0073 | 0.0583 | 9 | 0.0101 | 0.0054 | 0.0004 | 0.0227 |
| Sequence | CpGperCpG | 8 | 0.0149 | 0.0076 | 0.0038 | 0.0394 | 12 | 0.0066 | 0.0043 | 0.0002 | 0.0206 |
| Sequence | CpGobsExp | 11 | 0.0111 | 0.0046 | 0.0002 | 0.0227 | 11 | 0.0081 | 0.0048 | 0.0004 | 0.0226 |
| Sequence | GCContent | 26 | 0.0006 | 0.0004 | -0.0001 | 0.0017 | 23 | 0.0009 | 0.0005 | 0.0000 | 0.0024 |

## Supplementary Figures

| (a) | **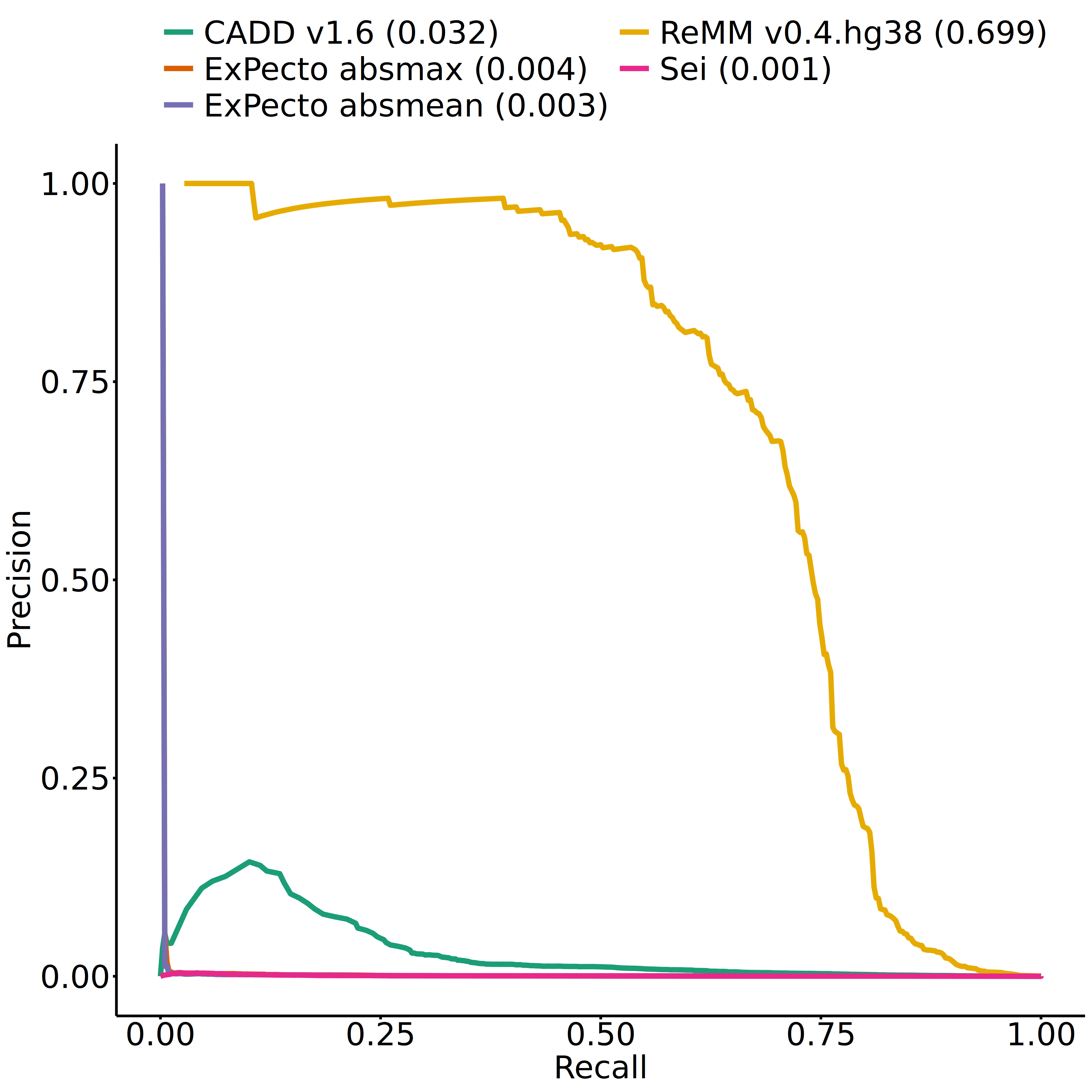** | (b) | **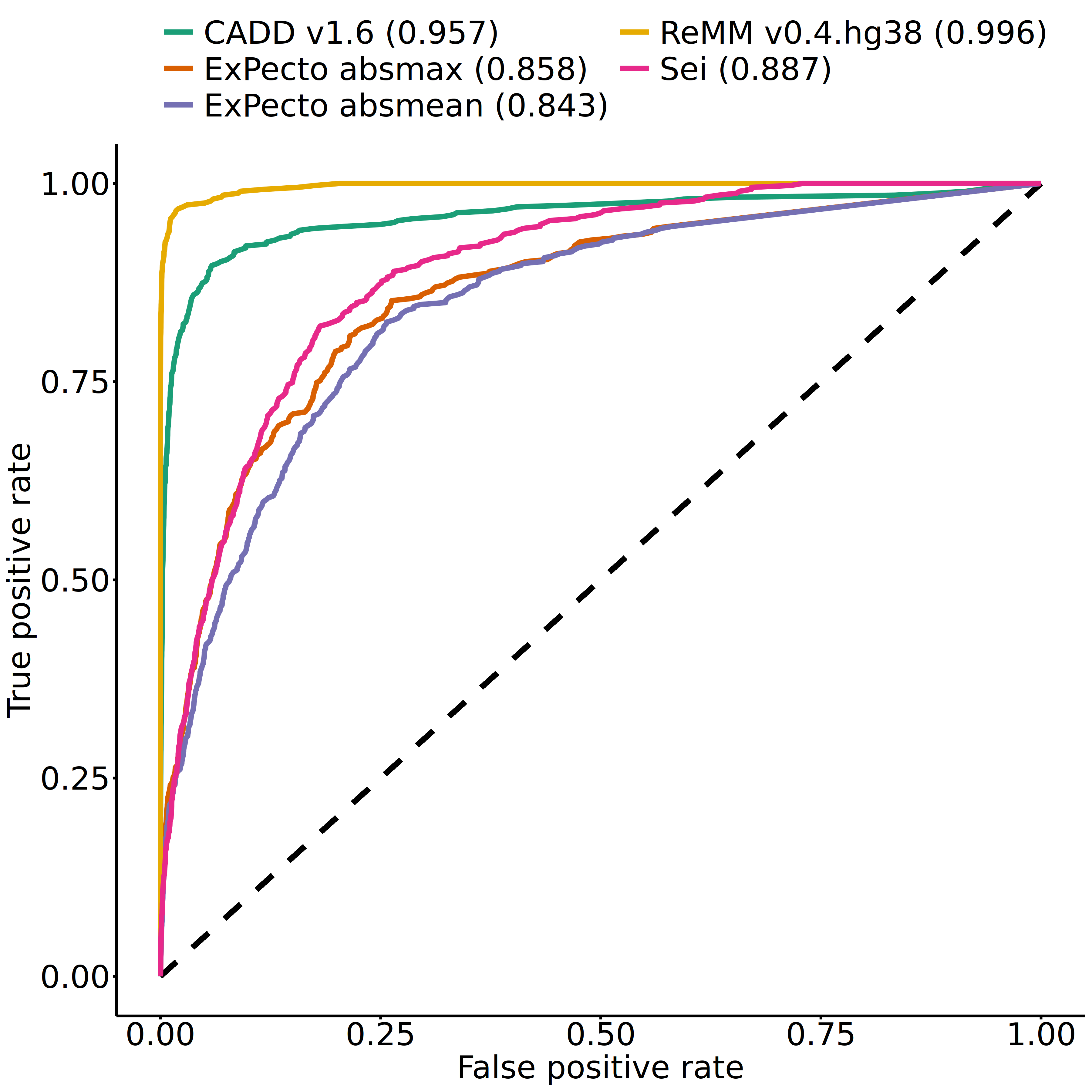** |
| --- | --- | --- | --- |

Supplementary Figure 1: ROC and PR curve of ReMM, CADD, ExPecto, and Sei for intersection of variants, scored by all tools – Precision-Recall (PR) curves (a) and receiver operating characteristic (ROC) curves (b) of ReMM v0.4.hg38 (ten-fold cytoband cross validation scores) as well as CADD v1.6, ExPecto, and Sei on the subset of the GRCh38 training data scored by all tools. In total 406 pathogenic and 7,299,993 proxy-benign variants were scored. Area under the curve is shown in parentheses. ExPecto absmax is the maximum absolute value over all ExPecto outputs and ExPecto absmean the mean absolute value, respectively.
