# Supplementary material for: The Regulatory Mendelian Mutation score for GRCh38
Source: Gigascience. 2023 Apr 21;12:giad024. doi: 10.1093/gigascience/giad024 (PMC10120424; doi:10.1093/gigascience/giad024)
Supplement: giad024_Response_to_Reviewer_Comments_Original_Submission [file giad024_response_to_reviewer_comments_original_submission.pdf]

Author's Response To Reviewer Comments

Close

We thank all reviewers and editor for their kind and constructive feedback on our manuscript. We addressed the additional comments brought up by the reviewers as well as the further requirements mentioned by the editor. We address each point in this response individually. Sections altered in the manuscript are highlighted in this document.

=====================================
Comments and tasks assigned by the editor
=====================================

* (editor) please register any new software application in the bio.tools and SciCrunch.org databases to receive RRID (Research Resource Identification Initiative ID) and biotoolsID identifiers, and include these in your manuscript. Computational workflows should be registered in workflowhub.eu and the DOIs cited in the relevant places in the manuscript. These will facilitate tracking, reproducibility and re-use of your tool.

(response) We have registered ReMM and provide its identifier (biotools:remm_score; RRID: SCR_023095; https://doi.org/ 10.48546/workflowhub.workflow.414.1) in the manuscript.

* (editor) Please include a point-by-point within the 'Response to Reviewers' box in the submission system. Please ensure you describe additional experiments that were carried out and include a detailed rebuttal of any criticisms or requested revisions that you disagreed with.

(response) We followed the editors' instructions, and this document provides the requested ‘Response to Reviewers’ document.

* (editor) Please also ensure that your revised manuscript conforms to the journal style, which can be found in the Instructions for Authors on the journal homepage. If the data and code has been modified in the revision process please be sure to update the public versions of this too.

(response) We made all required updated and we checked the author’s instructions again and believe that our manuscript matches all requirements.

==================
Reviewer comments
==================

Reviewer #1
----
1. (reviewer #1) In the abstract "Some methods and annotations are not available for the current human genome build (GRCh38), for which the adoption in databases, software and pipelines was slow." Not sure what the author is referring to by some methods, this could be a grammar problem.

(response) Thank you for this comment. We assume that the reviewer considers “methods” to be universal and independent of their exact implementation for a specific reference genome build, and that this causes some confusion. Here, we use “methods” as synonymous with “tools”. We modified the sentence to “Some tools for assessment of variant pathogenicity as well as annotations are not available for the current human genome build (GRCh38), for which the adoption in databases, software and pipelines was slow.” We hope this makes it clearer.

2. (reviewer #1) "Restricting variants to non-coding only removes a small proportion of variants", what is

the proportion? Also, I don't understand the need to remove coding variants, shouldn't your model works also with coding variants?

(response) The ReMM score as originally proposed by Smedley D et al. (Am J Hum Genet. 2016 Sep 1;99(3):595-606. doi: 10.1016/j.ajhg.2016.07.005) is a score for non-coding sequence variation. We remove the coding variants in the benign set to match the pathogenic training set which contains only non-coding variants. There is considerably more training data for coding variants, their inclusion in training would overshadow the small number of described pathogenic non-coding variants for which we aim to train a dedicated model. We added the proportion of dropped variants in the manuscripts (1% for GRCh37 and 0.7% for GRCh38).

3. (reviewer #1) The method the author used is based on a previous publication. However, there is still the need to give the detail of the method in this manuscript. There is a lot of missing information. For example, what is the outcome, whether a position is deleterious? How is the probability for deleteriousness calculated?

(response) Thank you for the feedback. Indeed, we were very short in our methods, with details only available from the respective references. This makes it difficult to read for anyone not familiar with the previous work. Therefore, we extended the method section and provide more information to the reader.
hyperSMURF/parSMURF:
“The hyperSMURF algorithm applies a special sampling technique essential for the highly imbalanced data of human pathogenic variants [1], [5]. The minority class (for ReMM the pathogenic variants) is oversampled based on the Synthetic Minority Over-sampling Technique (SMOTE) that creates synthetic examples using k-nearest neighbors rather than over-sampling the data with replacement [11]. The majority class (proxy-benign set) is divided into n non-overlapping partitions, which then are subsampled according to a ratio parameter. The minority class is oversampled by factor 2 and the majority class is undersampled by factor 3 which leads to the ration of pathogenic vs. benign variants of 2-3 in a more balanced dataset with around 2,000 datapoints. However, each balanced dataset alone provides insufficient coverage of the large data space of the majority class. That is why hyperSMURF applies an ensemble method: it divides the dataset into 100 partitions each containing all oversampled pathogenic and one partition of downsampled proxy-benign variants. On each partition, a random forest [12] is trained and the final pathogenicity score is the average over the 100 predictions. It ranges from 0 (not pathogenic) to 1 (pathogenic) and gives the probability values of a variant to belong to the pathogenic training data. Thus, the higher the score, the more likely that a variant at that position is pathogenic. We used parSMURF as implementation of hyperSMURF, a fast and highly scalable model training tool based on random forest algorithms [10].”
Ten-fold cytogenic band-aware cross-validation:
“To handle the local correlation structure in the genome, we apply ten-fold cytogenic band-aware cross-validation (CV) [1]. This is a stratified CV approach where each cytoband of the genome is associated to one out of ten folds. Folds are assigned to have a similar number of pathogenic variants, cytobands without pathogenic variants are randomly assigned to a fold. Proxy-benign variants are considered in the folds of their associated bands. Thereby gnomically proximal (i.e., same cytoband) pathogenic and proxy-benign variants are considered together, making it more challenging for the learner to discriminate between the two groups. Ten separate models are trained on nine folds and validated on the tenth fold. Unbiased predictions of variants contained in the training set can be performed with the model that only used the variants in the validation fold, while other variants are reported as the average of the ten model predictions.”

4. (reviewer #1) by a few specific variants. Thus, the overall Mendelian disease-related variants should be low. I am guessing that's why 406 hand-curated variants were used in the previous version of ReMM. If my assumption is correct, there shouldn't be a lot variants for Mendelian disease. How many variants are found to be positive in the entire genome?

(response) That is correct, the same pathogenic set was used for the training of the previous ReMM version. We also agree that we would not expect many Mendelian disease-related non-coding variants in the whole genome, however there is also not much empirical data to substantiate any specific claims. Using a mostly coding-based diagnostic approach, current studies identify disease-causing variants in up to

40% of large patient cohorts, leaving a substantial proportion of patients with a potential non-coding cause. As of Jan 2022, there were about 85,000 coding pathogenic/likely pathogenic variants reported in NCBI ClinVar for what accounts to about 1.5% of the genome. However, more than 366,000 variants were reported as variants of uncertain clinical significance for that same part of the genome. Large-scale efforts like TopMed/BRAVO or gnomAD cataloged about 800 million variants genome-wide, which is still a minority of all possible 9 billion single nucleotide changes to the human genome. Given all these considerations, it is unclear how many pathogenic variants ReMM should predict when applied to the whole genome.
To perform the analysis suggested by the reviewer, we counted all non-N positions in the human reference genome (GRCh38: 2,923,716,084, GRCh37: 2,862,362,542). We then determined those that are above the maximum F2 score (Figure 1 c and d). In total, the ReMM score predicts 25,734,725 pathogenic positions (0.9%) for GRCh37 and 37,888,567 positions (1.3%) for GRCh38. We note a considerable enrichment of coding regions in the predicted pathogenic positions (34%, n=8,957,838 for GRCh37/GENCODE v19); 35%, n=13,540,527 for GRCh38/GENCODE v42). It is likely that this is due to higher conservation scores in coding regions, another reason why we excluded coding effect in training and recommend that specialized coding scores should be used in this case.

5. (reviewer #1) In the online application, the results are limited to 500, the rest cannot be seen or downloaded. I would be better to allow the user to download the entire results.

(response) This comment probably refers to the range lookup (https://remm.bihealth.org/range-lookup). Here, we limit the output to 500 because the result is directly returned to the user's web browser. This part of our website only allows the user to download or copy results printed on the page. Larger ranges will cause performance issues for the browser. In this case, provided whole genome wide scoring files (indexed for direct retrieval of results using htslib/tabix or similar tools) as well as the UCSC browser tracks are much more useful. To help the user to retrieve larger ranges of scores, we added a note to the website that states the range limit and points to the existing solutions when scores of larger regions are needed.

6. (reviewer #1) The authors performed comparison with other tools and generated ROC curve which is dependent on knowing the true positives. There is no description of the dataset that was used for the comparison. Did the authors make sure that the training variants is not used for the comparison?

(response) We added further score / tool comparisons (e.g., CADD, LINSIGHT, fathmm-MKL, NCBoost, Expecto, Sei, RegBase) on a validation set based on GRCh37 NCBI ClinVar variants, for which any overlap with the training data set has been excluded. New plots can be found in the manuscript as Figure 4a and b. We further added a section in the methods with the description of the ClinVar dataset. We copy the text here:
"Due to the very limited availability of non-coding scores on GRCh38, we compared ReMM on GRCh37 with multiple other scores and on a set of non-coding variants from NCBI ClinVar that do not overlap its training set. We only used variants where all scores were able to provide a prediction (869 pathogenic and 190,548 benign) and plotted PR and ROC curves (Figure 4a and Figure 4b). CADD v1.6 achieved the best performance in terms of AURPC (0.160) and area under the ROC curve (AUROC, 0.811) followed by the (liftover) GRCh38 version of ReMM (AUPRC=0.035, AUROC=0.694). Interestingly, CADD v1.3, a previous version that does not yet include features for intronic splice variants, has a much lower performance on the non-coding ClinVar dataset (AUPRC=0.012, AUROC=0.633). We therefore speculate, that the performance boost in CADD v1.6 is due to the presence of many splice variants in the non-coding ClinVar dataset and the inclusion of specific splice-scores, like SpliceAI [33] or MMSsplice [34], in recent CADD versions. All compared scores, excluding CADD v1.6, are not optimized for splicing effects. Further, ReMM's pathogenic training set does neither contain splice variants nor did we add specific splicing features with this update."

Reviewer #2
----
1. (reviewer #2) How to deal with missing value variants in test datasets when compare new ReMM with other tools, the author mentioned that ExPecto annotated only half of the million negative variants.

(response) Yes, ExPecto was only able to predict 7 million variants of our proxy benign set. For all performance metrices/curves, we used all data that a score was able to predict. Therefore, performance

measurements of ExPecto were only done on half of the proxy benign set. We had already mentioned in the manuscript that this strategy might overestimate performance for scores with a lot of missing values. A second option would be using all not predictable variants as false classifications, which we considered an overly harsh approach. As a third option, comparison could be limited to (a potentially small intersection of) variants for which all tools are able to produce predictions. We now include a figure showing the comparison on the intersection of variants in the supplement (Supplementary Figure 1). Order and general results are stable despite the difference in the number of variants scored.

2. (reviewer #2) Although the CADD used the same negative training dataset, it's not suitable to compare it in the ReMM training dataset. How those tools performance in the independent test datasets.

(response) We agree that training datasets and objectives of CADD and ReMM are very different, and that the training performance should not be compared. This was also not our intention. ReMM uses a manually curated pathogenic set and contrasts that with an evolutionary derived benign variant set. This happens to be part of the CADD training dataset, but CADD uses it in a surrogate training approach (with substantial mislabeling and low accuracy on its training data) where random variants are contrasted with variants that have seen many generations of purifying selection to derive a measure of deleteriousness. Our intention was to analyze whether CADD can identify the manually curated pathogenic variants that ReMM uses for its training. Focusing on precision-recall, it turns out that it does so better than some state-of-the-art sequence-based predictors. We agree that this analysis has its limitations, due to the overlap of the training datasets as the reviewer(s) point out, but with CADD’s own training accuracy of only 62.2% (GRCh38-v1.6), it is also not as circular as one might think. Further, we are using the cross-validation-derived ReMM scores. However, to make an unbiased comparison we added a comparison on the NCBI ClinVar set of pathogenic and benign variants that were not present in any training set (see response reviewer #1, point 6).

3. (reviewer #2) The author presumes that new genome build will get better performance, is there some evidence can support this perspective, like the distribution of feature or training data in different genome build.

(response) The new genome build closed over 100 gaps and increased the number of resolved bases (e.g., 2,923,716,084 non-N positions in GRCh38 vs 2,862,362,542 non-N positions in GRCh37). This means that features based on sequence comparisons, like species conservation or chromatin assays, can be expected to be more accurate. For example, for the 26 features of our pathogenic variant set (n=406), the number of missing values reduced from 2,390 for GRCh37 to 2,154 for GRCh38 (proxy-benign from 140,548,778 down to 131,133,851). Similarly, on the >100k positions (i.e., positions with reciprocal liftover used in our correlation analysis between the genome builds), we see a reduction in missing values from 1,071,510 to 1,067,415. This allows the model a better distinction between the two class labels. We can also show this effect using a Wilcoxon Rank Sum (WRS) test between the two class labels for all individual features. While for GRCh38 all but one feature (DGVCount_20200225) shows a significant shift between the labels, for GRCh37 four features show a non-significant shift (Fantom5Perm, Fantom5Robust, DGVCount_20200225, rareVar).

4. (reviewer #2) Other existing similar tools can prioritization disease-causal noncoding variant, such as regBase-PAT, NCBoost, ncER, etc. can the authors compare new version of ReMM with these tools.

(response) We agree that there are other scores that can be used to prioritize non-coding variants. But unfortunately, all scores are limited to the GRCh37 genome release, limiting their use in new sequencing projects in clinic and research. Coordinate liftover can be performed between builds but has clear limitations when it comes to newly assembled regions and (depending on use) might be insensitive to changes in the exact sequence. This is one of the main motivations to build a new ReMM score for the GRCh38 release. To perform a sensible comparison, we decided to lift the GRCh38 score to GRCh37 and provide a comparison with several of the state-of-the-art non-coding scores (i.e., CADD, LINSIGHT, fathmm-MKL, NCBoost, Expecto, Sei, RegBase) on a ClinVar benchmark (Figure 4). We stress however, that the number of scores that can be applied to GRCh38 without liftover is very limited.

Reviewer #3

(reviewer #3) This is a valuable resource for the community of researchers and clinicians working on the interpretation of genetic variants in the human genome. The work appears to be thoughtfully done and appropriate assessments have been provided. The use of the random forest models to weigh the contributions of features was particularly noted for the insights it provided into how features contribute to prediction.

(response) Thank you for the kind review of our work.

(reviewer #3) My biggest concerns are stylistic, which falls outside the scientific quality of the work. I provide these comments for the authors to consider and do not expect that my stylistic preferences will be uniformly accepted. A fair amount of justification of the manuscript focuses on the value of having a release for version 38 of the human genome, pointing to the field as not having done so broadly. I think this is misguided, as by the time people are reading the manuscript such points will have lost relevance. I suggest a focus on the science be given, as there is no need to justify things based on where other resources have progressed in releasing their version 38 updates. Points below include language/text clarifications that can be assessed by the authors. Writing styles differ, so stylistic comments should be optional.

(response) Thank you for this insightful comment. We are a little torn here. We feel that our critique of a field that has very slowly adapted to GRCh38 and that keeps arguing with the small number of sequence changes as a reason to not adjust tools and pipelines is still very timely. Recent publications of the Telomere-to-Telomere (T2T) consortium have been very clear about the effects of missing genes, incorrect assemblies and artifacts of aligning to an incomplete reference. We believe that there might be some critical progress on the horizon. We also understand that our criticism will be dated quickly. However, each publication has its historic context in which it was written and ours has been written with some level of frustration over the adoption of GRCh38 in the community. Anyways, we thank the reviewer for that valuable and positive feedback on our work. We went through your minor points and incorporated your suggestions. Please see below for a detailed response.

1. (reviewer #3) The word "various" is vague and often shows up when people are too busy to provide an accurate statement. Starting the manuscript with it makes a bad impression on this reader. You do not have to change it, but I thought you might appreciate knowing this impression. You could delete it with no harm to the sentence. (Not to get carried away, but the next sentence starting with "some" heightens the impression of 'hand waving'.)

(response) Thank you! Yes, we appreciate your clarification here. We deleted “various” in the abstract and are not using it throughout the manuscript. We will consider its usage carefully in future manuscripts.

2. (reviewer #3) I think I understand ", we apply cytogenic band-aware cross-validation using ten folds" but I encourage the authors to provide clearer wording for this point.

(response) We revised the method section and added a paragraph explaining the cytogenic band-aware cross-validation as outlined above.

3. (reviewer #3) I would allow the reader to make their own judgement of performance. So please remove "excellent" from "we achieve an excellent performance"
The word “excellent” was removed from the manuscript.

4. (reviewer #3) "Rather than using ReMM scores for ranking, some users need to specify score thresholds" is confusing. I would change 'need to' to 'choose to'

(response) We changed “need to” to “choose to”.

5. (reviewer #3) "with lots of false positives" is a bit informal. I suggest "with a high false positive rate"

(response) We modified it as suggested.

6 (reviewer #3) I am confused by "from three genomic regions (genic content and not overlapping with assembly gap changes) " as the brackets include two items, not three.

(response) We modified the section to include more details which were only available in the supplement. The new section reads as follows:
“To compare both genome builds, we correlate ReMM scores from three genomic regions without assembly gap changes (DLK1, HBB, and PRDM9 loci) and >100,000 randomly sampled autosomal positions with successful reciprocal liftover (Supplementary Table 4). Here, ReMM scores are highly correlated between versions (Spearman and Pearson correlation between 0.7 and 0.8, Supplementary Table 4). We also used these regions and sites to explore the average feature correlation and find those to be similar, but except for one region (PRDM9) to be lower (Spearman correlation of 0.7 and Pearson correlation between 0.6-0.8, Supplementary Table 5).”

7. (reviewer #3) "maybe due to better mapping" - "maybe" should be "may be"

(response) Thank you. We corrected it.

8. (reviewer #3) I think the language like "seems to be the only tool directly trained on training data and features derived from GRCh38." Is not particularly valuable long term. This is a useful contribution, but many tools are being updated to 38 and by the time this appears and is read, such statements decline in relevance. I would focus on providing this valuable resource, and not try to justify it based on a transient perception of where the field stands in updating versions.

(response) We agree, but there is considerable hesitation to update or to even develop new tools on the current reference genome. We rephrased to make clear that 9 years after the GRCh38 release, only one score got updated on GRCh38 and that no other score exists for a direct comparison on GRCh38:
“However, a majority of tools are still based on the GRCh37 genome build which comes with the previously discussed drawbacks when scores are lifted to a new genome build[3], [4], [31]. To our knowledge, CADD, one of the most popular whole genome scores, seems to be the only tool directly trained on training data and features derived from GRCh38. Nine years after the GRCh38 release, no other non-coding score is adapted to the new genome build. Also, more recent sequence-based tools, like ExPecto [16] or DeepSEA-Sei [17] are trained on the previous genome release.”

9. (reviewer #3) "It is worth noting that in the context of extremely unbalanced data…" - you do note it. So I would change the wording to "In the context of extremely unbalanced data…"

(response) Thank you. We corrected that.

Close
