# Supplementary material for: The Regulatory Mendelian Mutation score for GRCh38
Source: Gigascience. 2023 Apr 21;12:giad024. doi: 10.1093/gigascience/giad024 (PMC10120424; doi:10.1093/gigascience/giad024)
Supplement: giad024_Reviewer_1_Report_Original_Submission — Yan Guo -- 10/11/2022 Reviewed [file giad024_reviewer_1_report_original_submission.pdf]

Reviewer Report

Title: The Regulatory Mendelian Mutation score for GRCh38

Version: Original Submission **Date:** 10/11/2022

Reviewer name: Yan Guo

Reviewer Comments to Author:

1. In the abstract "Some methods and annotations are not available for the current human genome build (GRCh38), for which the adoption in databases, software and pipelines was slow." Not sure what the author is referring to by some methods, this could be a grammar problem.2. "Restricting variants to non-coding only removes a small proportion of variants", what is the proportion? Also, I don't understand the need to remove coding variants, shouldn't your model works also with coding variants?3. The method the author used is based on a previous publication. However, there is still the need to give the detail of the method in this manuscript. There is a lot of missing information. For example, what is the outcome, whether a position is deleterious? How is the probability for deleteriousness calculated?4. by a few specific variants. Thus, the overall Mendelian disease-related variants should be low. I am guessing that's why 406 hand-curated variants were used in the previous version of ReMM. If my assumption is correct, there shouldn't be a lot variants for Mendelian disease. How many variants are found to be positive in the entire genome?5. In the online application, the results are limited to 500, the rest cannot be seen or downloaded. I would be better to allow the user to download the entire results.6. The authors performed comparison with other tools and generated ROC curve which is dependent on knowing the true positives. There is no description of the dataset that was used for the comparison. Did the authors make sure that the training variants is not used for the comparison?

Level of Interest

Please indicate how interesting you found the manuscript: Choose an item.

Quality of Written English

Please indicate the quality of language in the manuscript: Choose an item.

Declaration of Competing Interests

Please complete a declaration of competing interests, considering the following questions:

- Have you in the past five years received reimbursements, fees, funding, or salary from an organisation that may in any way gain or lose financially from the publication of this manuscript, either now or in the future?

- Do you hold any stocks or shares in an organisation that may in any way gain or lose financially from the publication of this manuscript, either now or in the future?
- Do you hold or are you currently applying for any patents relating to the content of the manuscript?
- Have you received reimbursements, fees, funding, or salary from an organization that holds or has applied for patents relating to the content of the manuscript?
- Do you have any other financial competing interests?
- Do you have any non-financial competing interests in relation to this paper?

If you can answer no to all of the above, write 'I declare that I have no competing interests' below. If your reply is yes to any, please give details below.

I declare that I have no competing interests

I agree to the open peer review policy of the journal. I understand that my name will be included on my report to the authors and, if the manuscript is accepted for publication, my named report including any attachments I upload will be posted on the website along with the authors' responses. I agree for my report to be made available under an Open Access Creative Commons CC-BY license (http://creativecommons.org/licenses/by/4.0/). I understand that any comments which I do not wish to be included in my named report can be included as confidential comments to the editors, which will not be published.

Choose an item.

To further support our reviewers, we have joined with Publons, where you can gain additional credit to further highlight your hard work (see: https://publons.com/journal/530/gigascience). On publication of this paper, your review will be automatically added to Publons, you can then choose whether or not to claim your Publons credit. I understand this statement.

Yes Choose an item.
