# Supplementary material for: The Regulatory Mendelian Mutation score for GRCh38
Source: Gigascience. 2023 Apr 21;12:giad024. doi: 10.1093/gigascience/giad024 (PMC10120424; doi:10.1093/gigascience/giad024)
Supplement: giad024_Reviewer_3_Report_Original_Submission — Wyeth Wasserman -- 11/22/2022 Reviewed [file giad024_reviewer_3_report_original_submission.pdf]

Reviewer Report

Title: The Regulatory Mendelian Mutation score for GRCh38

Version: Original Submission **Date:** 11/22/2022

Reviewer name: Wyeth Wasserman

Reviewer Comments to Author:

SYNOPSISThe manuscript describes an updated release of the ReMM regulatory variant mutation scoring system. The paper presents the performance of an updated version of the system and describes how it was applied to the most current release of the reference human genome.OVERALL PERSPECTIVEThis is a valuable resource for the community of researchers and clinicians working on the interpretation of genetic variants in the human genome. The work appears to be thoughtfully done and appropriate assessments have been provided. The use of the random forest models to weigh the contributions of features was particularly noted for the insights it provided into how features contribute to prediction.My biggest concerns are stylistic, which falls outside the scientific quality of the work. I provide these comments for the authors to consider and do not expect that my stylistic preferences will be uniformly accepted. A fair amount of justification of the manuscript focuses on the value of having a release for version 38 of the human genome, pointing to the field as not having done so broadly. I think this is misguided, as by the time people are reading the manuscript such points will have lost relevance. I suggest a focus on the science be given, as there is no need to justify things based on where other resources have progressed in releasing their version 38 updates. Points below include language/text clarifications that can be assessed by the authors. Writing styles differ, so stylistic comments should be optional.MAJOR POINTSNone. Well done and clearly presented.MINOR POINTS1. The word "various" is vague and often shows up when people are too busy to provide an accurate statement. Starting the manuscript with it makes a bad impression on this reader. You do not have to change it, but I thought you might appreciate knowing this impression. You could delete it with no harm to the sentence. (Not to get carried away, but the next sentence starting with "some" heightens the impression of 'hand waving'.)2. I think I understand ", we apply cytogenic band-aware cross-validation using ten folds" but I encourage the authors to provide clearer wording for this point.3. I would allow the reader to make their own judgement of performance. So please remove "excellent" from "we achieve an excellent performance"4. "Rather than using ReMM scores for ranking, some users need to specify score thresholds" is confusing. I would change 'need to' to 'choose to'5. "with lots of false positives" is a bit informal. I suggest "with a high false positive rate"6. I am confused by "from three genomic regions (genic content and not overlapping with assembly gap changes) " as the brackets include two items, not three.7. "maybe due to better mapping" - "maybe" should be "may be"8. I think the language like "seems to be the only tool directly trained on training data and features derived from GRCh38." Is not particularly valuable long term. This is a useful contribution, but many tools are being updated to 38 and by the time this appears and is read, such statements decline in relevance. I would focus on providing this valuable resource, and not try to justify it based on a transient perception of where the field stands

in updating versions.9. "It is worth noting that in the context of extremely unbalanced data…" - you do note it. So I would change the wording to "In the context of extremely unbalanced data…"

Level of Interest

Please indicate how interesting you found the manuscript: Choose an item.

Quality of Written English

Please indicate the quality of language in the manuscript: Choose an item.

Declaration of Competing Interests

Please complete a declaration of competing interests, considering the following questions:

- Have you in the past five years received reimbursements, fees, funding, or salary from an organisation that may in any way gain or lose financially from the publication of this manuscript, either now or in the future?
- Do you hold any stocks or shares in an organisation that may in any way gain or lose financially from the publication of this manuscript, either now or in the future?
- Do you hold or are you currently applying for any patents relating to the content of the manuscript?
- Have you received reimbursements, fees, funding, or salary from an organization that holds or has applied for patents relating to the content of the manuscript?
- Do you have any other financial competing interests?
- Do you have any non-financial competing interests in relation to this paper?

If you can answer no to all of the above, write 'I declare that I have no competing interests' below. If your reply is yes to any, please give details below.

I have no competing interests.

I agree to the open peer review policy of the journal. I understand that my name will be included on my report to the authors and, if the manuscript is accepted for publication, my named report including any attachments I upload will be posted on the website along with the authors' responses. I agree for my report to be made available under an Open Access Creative Commons CC-BY license (http://creativecommons.org/licenses/by/4.0/). I understand that any comments which I do not wish to be included in my named report can be included as confidential comments to the editors, which will not be published.

Choose an item.

To further support our reviewers, we have joined with Publons, where you can gain additional credit to further highlight your hard work (see: https://publons.com/journal/530/gigascience). On publication of this paper, your review will be automatically added to Publons, you can then choose whether or not to claim your Publons credit. I understand this statement.

Yes Choose an item.
